# Terpenoids as Emerging Senotherapeutics: Mechanistic Insights and Therapeutic Potential

**DOI:** 10.3390/ijms27156874

**Published:** 2026-07-31

**Authors:** Sungwoo Yi, Jung Yoon Park, Sung-Joon Lee

**Affiliations:** 1 Department of Biotechnology, Graduate School of Biotechnology, Korea University, Seoul 02841, Republic of Korea; liveforever@korea.ac.kr (S.Y.); gabriella810@korea.ac.kr (J.Y.P.); 2 Department of Food Bioscience and Technology, College of Life Sciences and Biotechnology, Korea University, Seoul 02841, Republic of Korea; 3 Interdisciplinary Program in Precision Public Health, BK21 Four Institute of Precision Public Health, Korea University, Seoul 02841, Republic of Korea

**Keywords:** aging, cellular senescence, terpenoid, senotherapeutics, natural compounds

## Abstract

Cellular senescence, characterized by stable cell cycle arrest, drives organismal aging and functional decline. Senescent cells (SnCs) increase in multiple tissues with age and contribute to pathology by resisting apoptosis, impairing regeneration, and releasing senescence-associated secretory phenotype (SASP) factors. Senotherapeutics, including senolytics and senomorphics, aim to reduce the impact of SnCs by selectively clearing SnCs or suppressing SASP production. Natural terpenoids are structurally diverse plant- and fungus-derived metabolites with antioxidant, anti-inflammatory, and cytoprotective properties. Growing evidence supports their ability to counteract senescence-associated phenotypes, including senescence-associated β-galactosidase (SA-β-gal) activity and the expression of p53, p21, and p16. In this review, we examine mono-, sesqui-, di-, and triterpenoids that modulate cell cycle regulation, SASP attenuation, redox homeostasis, mitochondrial function, autophagy, and apoptosis-related mechanisms. We also outline the principal pathways underlying these anti-senescence activities and highlight natural terpenoids as emerging modulators of cellular senescence.

## 1. Introduction

Cellular senescence refers to persistent and irreversible cell cycle arrest triggered by diverse forms of cellular stress, accompanied by macromolecular damage, secretory features, and altered metabolism [1]. Cellular senescence is typically promoted by multiple factors, including DNA damage, oxidative stress, and ionizing radiation [2], and is widely recognized as a hallmark of organismal aging that contributes to the decline in physical function [1,3].

During aging, senescent cells (SnCs) progressively accumulate across multiple organs. SnCs adopt an enlarged and flattened shape, often displaying an accumulation of lysosomal and mitochondrial contents [4]. Beyond their morphological changes, SnCs exhibit a pro-inflammatory secretory profile known as the senescence-associated secretory phenotype (SASP) [5]. SASP factors comprise not only inflammatory cytokines and chemokines but also enzymatic regulators, such as matrix metalloproteinases (MMPs) and serine/cysteine proteinase inhibitors (SERPINs) [5]. This secretory repertoire is highly context-dependent, with its molecular signatures tailored to the cell type and stress source.

Sustained secretion of SASP molecules induces inflammaging, which disrupts tissue remodeling, the immune system, and homeostatic control [6]. Furthermore, once stem cells and progenitor cells undergo cell cycle arrest, they fail to replenish damaged tissues, thereby impairing the regenerative capacity of the organs [5]. Thus, cellular senescence plays a leading role in the onset and progression of various age-related diseases, such as cardiovascular disease, obesity, type 2 diabetes, pulmonary fibrosis, and sarcopenia [7,8].

Notably, genetic or pharmacological clearance of SnCs significantly enhanced physical function and extended lifespan in aged mice [9,10,11,12,13]. Hence, therapeutic interventions against SnCs have gained prominence as a compelling strategy for retarding aging and delaying the onset of age-related pathologies. Senotherapeutics are agents that target and eliminate or modulate SnCs to mitigate the aging process. They are categorized based on their strategy: senolytics focus on the selective elimination of SnCs through apoptosis, whereas senomorphics/senostatics aim to neutralize the SASP without inducing cell death [14,15].

To validate whether senotherapeutics attenuate the SnCs phenotype, various cellular senescence markers have been identified [2,4,15]. SnCs universally exhibit elevated activity of senescence-associated beta-galactosidase (SA-β-gal), the most widely used biomarker of senescence. Cell cycle inhibitors, including p53, p21^WAF1/CIP1^, p16^INK4a^, and RBs, are established molecular markers that directly induce and maintain cellular senescence. In contrast, SnCs feature a lack of Ki67 and PCNA, the cell proliferation markers. In addition, SnCs harbor unrepaired DNA damage, which is identified by distinct foci arising from the accumulation of phosphorylated H2AX (γ-H2AX), 53BP1, and MDC1 at the damage sites. The loss of Lamin B1, a protein that supports the nuclear envelope, is also a key characteristic of aged cells. Senescence status can be evaluated by measuring SASP factors at the mRNA and protein levels as well [2,4,15].

While SnCs maintain survival even in damaged or harsh environments, targeting the specific mechanisms that allow them to evade apoptosis can exert potent senolytic activity. Senolytics targeting antiapoptotic pathways, specifically through the inhibition of the BCL-2 family (ABT-263 and ABT-737) [16,17], p53/MDM2 (UBX0101 and RG7112) [18,19], and HSP90 (17-DMAG, 17-AAG, and geldanamycin) [20] have been discovered. Meanwhile, compounds such as metformin [21], rapamycin [22], and sirtuin activators [23] have been reported to exhibit senomorphic effects by downregulating p38 MAPK, NF-κB, JAK/STAT, and mTOR signaling pathways [14], which are key factors driving excessive SASP production.

Conventional small-molecule senolytic drugs have often been hindered in their clinical application by systemic toxicity and adverse effects. For example, ABT-263 (navitoclax) may trigger severe thrombocytopenia [24], and patients with idiopathic pulmonary fibrosis treated with dasatinib and quercetin (D + Q) exhibited a higher incidence of mild side effects (unwellness, cough, nausea, fatigue, weakness, and headache) than those in the placebo group [25]. Considering these limitations, the use of natural compounds as senotherapeutics has emerged as a promising intervention to counteract the senescent cell burden and its pathological consequences.

Phytochemicals with senotherapeutic activity have been extensively explored in polyphenolic classes, including fisetin [26], quercetin [27], EF-24 (a curcumin analog) [28], o-vanillin [19], kaempferol [29], apigenin [29], and luteolin [30]. In this context, multiple reviews have reported the senolytic or senomorphic activities of polyphenols [31,32,33]. In contrast, terpenoids remain relatively underexplored as senotherapeutics; however, their potential anti-senescence activity may be inferred from mechanistic parallels with polyphenols [34,35]. Accumulating evidence indicates that terpenoids can modulate the core features of senescence, including cell cycle arrest, SASP production, mitochondrial dysfunction, and oxidative stress [36]. Accordingly, a dedicated review integrating evidence across terpenoid subclasses, senescence models, and molecular pathways is warranted to define the senotherapeutic relevance of the relatively underexplored compound class. In this review, we aimed to delineate the current findings to define the anti-senescence potential of terpenoids and provide a mechanistic framework for their action as senotherapeutic candidates.

## 2. Senotherapeutic Effects of Terpenoids

Terpenoids, chemically diverse compounds, represent a large class of plant secondary metabolites. They are biosynthesized from five-carbon isoprene units and can be commonly classified as mono-(C10), sesqui-(C15), di-(C20), and tri-(C30) terpenes, based on their carbon number [37]. Although terpenoids originate from simple units, their structures are highly diverse due to elongation and cyclization. The structural diversity of terpenoids enables them to interact with multiple molecular targets, contributing to their wide range of biological activities [38]. Ample evidence from both in vitro and in vivo studies has shown that terpenoids can alleviate cellular senescence. This section provides a comprehensive overview of terpenoids in aging, with a particular focus on cellular senescence. The effects of terpenoids are pleiotropic, and they regulate senescence markers both directly and indirectly (Table 1).

### 2.1. Monoterpenoids

#### 2.1.1. Limonene

Limonene is a monocyclic monoterpenoid abundant in the essential oils of citrus fruits and other aromatic plants. Owing to its high-quality fragrance, limonene is widely used as an additive in food and cosmetic product formulations. In addition, it possesses diverse bioactive properties, including anti-inflammatory, antioxidant, antitumorigenic [98], and neuroprotective effects [39]. In particular, the robust radical-scavenging activity of D-limonene confers its potential as a preventive and therapeutic agent, particularly for skin aging.

**Figure 1:**
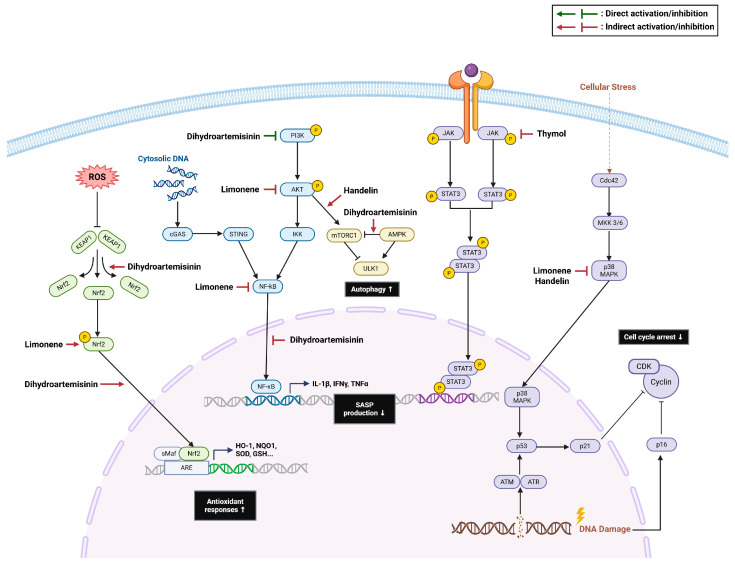
Major schematic pathways of senotherapeutic monoterpenoids and sesquiterpenoids. Monoterpenoids and sesquiterpenoids exert senotherapeutic effects through multiple pathways. This schematic depicts four molecular pathways commonly involved in these effects. Antioxidant pathways are shown in light green, anti-inflammatory pathways in blue, autophagy-related pathways in yellow, and senescence regulatory pathways in purple. Limonene and dihydroartemisinin activate Nrf2 antioxidant signaling [39,44,47] while suppressing PI3K/AKT [39,45] and NF-κB pathway [40,45], thereby reducing SASP production. Dihydroartemisinin also exhibits senolytic activity through AMPK activation-mediated autophagy and subsequent ferroptosis [43]. Thymol inhibits JAK1/STAT3 signaling [42], and handelin attenuates senescence through enhancing AKT/mTORC1 signaling [49] and suppressing p38 MAPK phosphorylation [48]. The depicted pathways were identified in distinct senescence models and should not be interpreted as a single unified mechanism operating simultaneously within one biological system. Rather, they represent context-specific mechanisms that differ across cellular environments and experimental settings. Arrows indicate pathway activation, whereas blunt-ended lines indicate pathway inhibition. Green and red arrows denote direct molecular binding and indirect modulation, respectively. Created in BioRender. Lee, S. (2026) https://BioRender.com/ee0hrte (accessed on 19 July 2026).

Limonene protects the skin from UVB exposure by reducing reactive oxygen species (ROS) generation. Pretreatment with limonene in human keratinocyte HaCaT cells activated PI3K/AKT signaling and induced nuclear translocation of Nrf2, thereby enhancing the Nrf2-mediated antioxidant defense system (Figure 1). Limonene also inhibited p53 phosphorylation, suppressed α-MSH secretion, and protected the skin barrier against photodamage [39]. In addition, limonene treatment counteracts the initiation and progression of aging-associated skin damage [40]. Six weeks of limonene treatment in a D-galactose-induced skin aging mouse model enhanced antioxidant defense by restoring superoxide dismutase (SOD) and glutathione peroxidase (GPx) activities, suppressed inflammation, and thereby attenuated epidermal thinning. The expression of pro-inflammatory cytokines, including TNF-α, IL-1β, and IL-6, was significantly reduced through the inhibition of NF-κB activation and p38 MAPK signaling [40]. Limonene also inhibited collagenase and elastase activities, which contribute to the loss of skin structural integrity and wrinkle formation, and suppressed MMP expression, thereby delaying skin aging [99].

Moreover, limonene has been reported to increase the lifespan of *C. elegans*. Limonene (5 μM) achieved maximum lifespan, increasing lifespan by 17.9%. It promoted longevity by scavenging ROS, activating DAF-16 (a FOXO ortholog), and upregulating stress resistance-related genes, such as *sod-3*, *daf-2*, and *hsp-70* [100]. Given that the DAF-16/FOXO transcription factor is evolutionarily conserved, evaluating the effects of limonene in more complex animal models would be of considerable interest and may provide further insight into its potential to promote longevity across species.

#### 2.1.2. Thymol, Carvacrol, Eugenol

Monoterpenoids exhibit a wide range of biological activities, particularly antioxidant and anticancer activities [101]. Among these, three essential oil–derived compounds, thymol, carvacrol, and eugenol, have recently emerged as potential senotherapeutics. Despite their structural differences, these compounds engage in somewhat similar mechanisms involving oxidative stress and apoptosis.

In H_2_O_2_-induced senescent human mesenchymal stromal cells (MSCs), thymol, carvacrol, and eugenol promoted senescent cell death without affecting healthy MSCs by triggering ROS-dependent mitochondrial apoptosis [35]. Thymol, carvacrol, and eugenol inhibited SRC signaling, which drives cell cycle progression from G1/G0 arrest to S phase and activates the G2/M transition, thereby shifting cell fate from senescence to cell death. In addition, these compounds increased the expression of pro-apoptotic factors, including BAX and cytochrome c, and enhanced intrinsic apoptotic signaling. Mechanistically, treatment with these compounds elevated intracellular ROS levels in SnCs beyond the level that can be effectively managed by the cellular antioxidant system, leading to destabilization of mitochondrial integrity [35]. Glutathione treatment attenuated this terpene-induced apoptosis, supporting the notion that oxidative stress is a key driver of SnCs elimination [35]. Although all three compounds achieve senolysis, they differ in the specific molecular mechanisms through which senescent cell death is induced. Carvacrol induces canonical caspase-dependent apoptosis, characterized by the activation of caspase-3, whereas thymol and eugenol rely on a caspase-independent pathway that induces nuclear translocation of apoptosis-inducing factor [35].

Beyond their senolytic activity, thymol exerts geroprotective effects. Thymol extended the healthspan of *C. elegans* and suppressed epigenetic aging in skeletal muscles [41]. Chronic treatment with thymol (20 mg/kg/day) for 12 weeks increased the myofiber cross-sectional area and improved physical performance in the SAMP8 mouse model, supporting its potential as a therapeutic candidate for preventing sarcopenia. Thymol also transiently reduced the mitochondrial membrane potential and induced a stress response, triggering a mitohormetic response through PINK-1 signaling [41]. In addition, thymol delays ovarian aging and preserves granulosa cell function by inhibiting JAK1/STAT3 signaling (Figure 1). Thymol inhibited JAK1 phosphorylation and reduced the expression of senescence markers p53, p21, and p16 in t-BHP-induced KGN cells. Moreover, thymol treatment increased estradiol levels and exerted beneficial effects on reproductive outcomes in aged mice [42]. Collectively, these findings position the herbal monoterpenes thymol, carvacrol, and eugenol as promising agents for mitigating cellular senescence.

### 2.2. Sesquiterpenoids

#### 2.2.1. Dihydroartemisinin

Dihydroartemisinin, the primary active metabolite of artemisinin, exhibits enhanced bioavailability and potency compared with its parent compound and has been widely investigated for its antimalarial, antitumor [102], antioxidant [103], and anti-inflammatory properties. Earlier studies revealed that dihydroartemisinin induces cancer cell death through ferroptosis [104], and subsequent studies have shown that this ferroptosis-promoting ability is also observed in SnCs, indicating that dihydroartemisinin functions as a senolytic agent. Dihydroartemisinin treatment in H_2_O_2_-induced senescent (SA-β-gal–positive) NIH3T3 cells activates ferroptosis, thus inducing selective cell death [43]. Mechanistically, dihydroartemisinin activates AMPK and suppresses mTOR phosphorylation, thereby promoting autophagy (Figure 1). Enhanced autophagy accelerates ferritin degradation and increases intracellular Fe^2+^ levels while concurrently inhibiting the key ferroptosis-protective system, Gpx4. Pharmacological inhibition of either autophagy or ferroptosis attenuated dihydroartemisinin-mediated clearance of SnCs, supporting the notion that dihydroartemisinin eliminates SnCs via autophagy-dependent ferroptotic mechanisms. SnCs exhibit elevated intracellular iron levels due to impaired iron homeostasis, rendering them more sensitive to dihydroartemisinin-dependent ferroptosis. In addition, treatment with dihydroartemisinin (50 mg/kg) extended the lifespan of naturally aged mice by 15.8%, further verifying its anti-aging effects in vivo [43].

Dihydroartemisinin has also been reported to alleviate cellular senescence across multiple tissues and exert protective effects against diverse diseases [44,45,46,47]. Dihydroartemisinin reversed myeloid-derived suppressor cells senescence through the upregulation of the Nrf2/HO-1 pathway and suppressed the progression of systemic lupus erythematosus, a chronic autoimmune disease [44]. In addition, dihydroartemisinin ameliorated TNFα–induced senescence in nucleus pulposus cells and improved extracellular matrix metabolism [45], supporting its therapeutic potential in intervertebral disc degeneration (IDD). Likewise, dihydroartemisinin improved Pfirrmann grade, which is a standard classification system used to evaluate the severity of IDD and delayed degenerative changes in a puncture-induced rat IDD model. Intraperitoneal administration of dihydroartemisinin at 40 mg/kg every other day significantly lowered the Pfirrmann score and restored the interrupted boundary between annulus fibrosus and nucleus pulposus [45]. These protective effects on IDD were mediated through the suppression of PI3K/AKT and NF-κB signaling (Figure 1). Dihydroartemisinin directly binds to PI3K and inhibits downstream pathways, thus attenuating the progression of IDD.

In an ischemia/reperfusion-induced renal aging model, dihydroartemisinin reversed premature senescence of renal tubular epithelial cells through the enhancement of autophagic activity and improved renal histopathology [46]. Dihydroartemisinin treatment significantly reduced SASP factors and alleviated necrosis and fibrosis progression in the kidney. It also increased the expression of Klotho, an anti-aging protein that counteracts the accumulation of SnCs [105]. Furthermore, dihydroartemisinin attenuated chondrocyte senescence and associated arthropathy both in vitro and in vivo, with chondroprotective effects mediated by the activation of the Keap1–Nrf2 axis [47]. Dihydroartemisinin induced the ubiquitination and degradation of Keap1 and increased the expression of antioxidant genes, thereby regulating cellular senescence.

Collectively, dihydroartemisinin exhibits multifaceted senotherapeutic effects across different models. While dihydroartemisinin selectively induces autophagy-dependent ferroptosis in senescent NIH3T3 cells, it attenuates senescence-associated phenotypes in renal, cartilage, and intervertebral disc degeneration models, thereby mitigating the development of age-related diseases. Given that dihydroartemisinin is an FDA-approved drug with low toxicity in normal cells and exhibits selective vulnerability to SnCs, dihydroartemisinin holds strong potential as a senolytic agent.

#### 2.2.2. Handelin

Handelin is a bioactive compound derived from *Chrysanthemum indicum* L. (wild chrysanthemum), which has traditionally been used to treat inflammatory diseases. A water extract of *C. indicum* L. was shown to attenuate UV irradiation-induced skin aging, and handelin was identified as one of the bioactive compounds mediating this protective effect. *C. indicum* L. extract significantly reduces ROS levels and protects HaCat cells from photoaging by inhibiting p38 MAPK activation (Figure 1) [48].

Handelin supplementation (50 μM) extended the lifespan of *C. elegans* by 19%, exhibiting longevity-promoting effects comparable to that of metformin [106]. It also improved motility and pharyngeal pumping, which are phenotypes for improving healthspan. These longevity effects are accompanied by a decline in intracellular ROS levels, and the gene expression of *gst-5*, *gst-33*, *stdh-2* and F12E12.11 was markedly upregulated following 8 days of handelin feeding [106]. The *gst-5* and *gst-33* are known to encode glutathione S-transferases and are involved in ROS detoxification, while F12E12.11 is predicted to exhibit NAD-dependent oxidoreductase activity. In addition, handelin contributes to the reversal of mitochondrial dysfunction. Handelin supplementation increased mtDNA copy number, improved mitochondrial morphology, and enhanced mitochondrial biogenesis during aging in worms [106]. Similarly, in 23-month-old mice, intraperitoneal administration of handelin for one month increased muscle redness and upregulated mitochondrial biogenesis genes, including *Pgc1α*, *Mfn1*, *Drp1*, and *Tfam1* [49].

Furthermore, handelin is effective in maintaining skeletal muscle integrity and preventing muscle atrophy. In TNF-α–treated C2C12 myotubes, handelin increased the expression of MyoD, myogenin, and myosin heavy chain, and directly enhanced myogenic differentiation by promoting the IGF-1/AKT pathway. Handelin also suppressed TNF-α-induced NF-κB activation, attenuated the inflammatory response, and consequently, mitigated muscle atrophy. These findings were further supported by in vivo evidence, as daily handelin administration increased skeletal muscle mass, downregulated *Fbxo32*, and restored the NAD level of tibialis anterior muscle in aged mice [49]. Notably, the protective effects of handelin against age-associated muscle dysfunctions were abolished by the Hsp70 inhibitor VER-155008, suggesting that Hsp70 mediates the beneficial effects of handelin on mitochondrial function and muscle health [49,107]. Combining these findings, Handelin delays aging through multiple mechanisms and may have therapeutic potential for aging-related conditions, including sarcopenia.

#### 2.2.3. β-Caryophyllene

β-Caryophyllene is a naturally occurring sesquiterpene found in the essential oils of various plants, including cinnamon, oregano, and black pepper. As a phytocannabinoid, it selectively activates cannabinoid receptor type 2 and exerts diverse bioactive properties. Beyond its anti-inflammatory, antioxidant, and anticancer properties [108,109,110], β-Caryophyllene has recently emerged as a potential geroprotective agent.

β-Caryophyllene treatment increased the lifespan of *C. elegans* by 22% while reducing intracellular ROS levels [50]. Mechanistic analyses revealed that the pro-longevity effects of β-Caryophyllene are mediated by *skn-1* and *sir-2.1*, corresponding to mammalian Nrf2 and SIRT1, respectively. Lifespan extension was abolished in *skn-1* and *sir-2.1* mutants but retained in *daf-16* mutants, indicating that β-Caryophyllene primarily engages in detoxification and stress resistance pathways rather than the canonical insulin/IGF-1 signaling. In addition, β-Caryophyllene reduced lipofuscin accumulation, which increases with aging, and decreased the pharyngeal pumping rate in worms, thereby promoting longevity in a dietary restriction (DR)-like manner [50].

Additionally, β-Caryophyllene restrained SASP expression in both replicative and drug-induced senescent human umbilical vein endothelial cells, as well as in LPS-induced THP-1 monocytes. While β-Caryophyllene alone effectively reduced the gene expression of pro-inflammatory cytokines, including *IL1B*, *IL6*, and *TNF*, and restored SIRT1 levels, this effect is synergistically enhanced when combined with resveratrol and curcumin. Furthermore, β-caryophyllene modulated microRNA expressions, downregulating *inflamma-miRs* (miR-146a, miR-21) and upregulating miR-126, which plays a critical role in maintaining vascular homeostasis [51]. These findings suggest that β-Caryophyllene suppresses inflammaging, which is exacerbated by the spread of inflammation to the surrounding tissues, and exerts a protective effect against endothelial senescence.

### 2.3. Diterpenoids

#### 2.3.1. Ginkgolide B

Ginkgolide B (GB), an active compound derived from *Ginkgo biloba*, is known to exert various health-promoting effects. GB has been reported to modulate inflammation and apoptosis and block oxidative stress [111], with accumulating evidence supporting its potential to promote longevity. Chronic oral administration (12 mg/kg) of GB prolonged the median lifespan of aged female mice (>20 months) by 8.5% and increased the maximal lifespan by approximately 50 days. In addition, GB markedly improved multiple healthspan parameters, including muscle strength, physical performance, frailty index, and metabolic indicators. At the molecular level, GB reduced the secretion of several SASP factors, including p16, p57, IL-6, and IFN-γ, in various tissues [52]. These findings suggest that Ginkgolide B exerts strong geroprotective effects.

Ginkgolide B also exerts a critical influence on muscle regeneration. In aged mice, GB treatment increased skeletal muscle mass and myosin heavy chain–positive area and reversed age-associated impairment of muscle function. Importantly, GB activated osteoblasts to restore osteocalcin levels to those observed in young mice and promoted muscle regeneration by stimulating the bone–muscle axis [53]. Endurance exercise in aged mice facilitates muscle repair by increasing IL-6 release from myocytes, which in turn enhances osteocalcin secretion. Similarly, GB enhanced osteocalcin–GPRC6A signaling and promoted myogenesis, supporting its potential role as an exercise mimetic [53]. Single-nucleus RNA sequencing analyses revealed that GB directly modulates aging-related pathways within the skeletal muscle and restores aging-associated transcriptional changes, including alterations in cell-type composition. GB downregulated Runx1, a factor that promotes cellular senescence through its interaction with p53, and reduced the formation of Runx1^+^ type 2B myonuclei, which accumulate with age [52]. Mechanistically, GB enhanced miR-27b-3p levels, thereby directly suppressing Runx1 and attenuating muscle degeneration.

These findings position Ginkgolide B as a promising senotherapeutic. GB effectively mitigates late-life aging and associated functional decline while preserving muscle integrity by restoring osteocalcin signaling. Given that musculoskeletal health is important for overall health and longevity in elderly individuals and is strongly associated with mortality, GB may provide a therapeutic avenue for promoting healthy aging.

#### 2.3.2. Oridonin

Oridonin, an active diterpenoid isolated from the medicinal herb *Rabdosia rubescens*, has been widely studied for its anti-inflammatory, antitumor, and antioxidant activities [112,113,114]. Oridonin was then identified as a novel senolytic compound through a screening of 18 bioactive ingredients of the Traditional Chinese Medicine library [54]. Senolytic activity was evaluated by comparing the viability of senescent and non-senescent cells using cisplatin-induced senescent A549 cells, and oridonin exhibited the most potent activity, with a ratio of 0.54. Activity-based protein profiling further identified GSTK1 as a direct target of oridonin [54]. Oridonin inhibits GSTK1 and increases cellular susceptibility to oxidative stress, leading to a marked increase in intracellular ROS levels. This, in turn, activates p38 signaling and downstream apoptotic pathways, ultimately promoting senolysis.

Effects of oridonin were further validated in various aging models. Oridonin attenuated SASP expression in bleomycin-induced BJ cells by inhibiting p38 phosphorylation and NF-κB p65 activity [55]. In doxorubicin-induced senescent WI-38 cells, oridonin significantly reduced the proportion of SA-β-gal-positive cells and cell cycle markers, in addition to SASP suppression [56]. In addition, monthly administration of oridonin extended the average lifespan of aged mice by 9.8% and improved both performance and memory function. These anti-aging effects are mediated by the AKT/FOXO1 pathway, as oridonin inhibits AKT activation and reduces p21 expression [56]. Altogether, these findings highlight the potential of oridonin as a senotherapeutic compound capable of targeting SnCs and mitigating age-related decline.

#### 2.3.3. Tanshinone IIA/Sodium Tanshinone IIA Sulfonate

Tanshinone IIA (TSA) is a diterpenoid quinone extracted from the medicinal herb *Salvia miltiorrhiza*, widely used to treat cardiovascular disorders. TSA is known to protect vascular function from inflammation and oxidative stress [115], and recent studies have demonstrated that TSA also exerts anti-senescent effects, particularly in hyperglycemic conditions. High glucose treatment accelerated senescence in human peritoneal mesothelial cells, whereas TSA counteracted this glucose-induced cellular aging, as evidenced by reduced SA-β-gal positivity and decreased p16 and p21 expression [57]. TSA has also been reported to alleviate H_2_O_2_-induced senescence in endothelial cells through the activation of SIRT1 [58].

Sodium tanshinone IIA sulfonate (STS), a water-soluble TSA derivative, was developed to improve its bioavailability and applicability. STS protects the vascular system from aging by targeting oxidative stress [59,60]. In high-glucose–treated endothelial cells and diabetic mouse models, STS markedly reduced intracellular ROS levels and decreased the number of SnCs [59]. In addition, STS reduced γ-H2AX and p21 expression while increasing CD31 and vWF levels in endothelial progenitor cells. These cellular effects are further translated into enhanced vascular repair and suppression of neointimal hyperplasia in diabetic mouse models [60]. The underlying mechanism of these effects involves the NF-κB–NLRP3 axis. STS inhibits the NLRP3 inflammasome by activating A20 and restores catalase activity, which is reduced under hyperglycemic conditions [59]. Taken together, TSA and its derivative STS attenuate cellular senescence induced by metabolic stress and provide a useful senotherapeutic strategy to counteract accelerated senescence associated with diabetes.

#### 2.3.4. Andrographolide

Andrographolide, a bioactive diterpenoid derived from *Andrographis paniculata*, possesses anti-inflammatory [116] and antioxidant [117] properties. Andrographolide has also been reported to exert protective effects against diabetic nephropathy by suppressing NF-κB activation and reducing oxidative stress [117].

Recent studies elucidated their roles in modulating cellular senescence. Andrographolide alleviated lipotoxicity-mediated premature senescence and mitigated renal fibrosis in HFD-fed mice [61]. Six weeks of andrographolide administration downregulated senescence markers, including p16, p21, and p53, and reduced ROS production and consequent oxidative DNA damage. Mechanistically, andrographolide acts as an AMPK agonist, and its AMPK activation activity reduced renal lipid accumulation and SASP formation, both of which are known to promote fibrogenesis. Moreover, andrographolide suppressed paracrine fibroblast activation [61]. SnCs can exacerbate fibrosis by stimulating neighboring fibroblasts, and this paracrine effect was effectively inhibited by andrographolide.

Andrographolide also inhibits senescence and exerts protective effects in the musculoskeletal system. In dexamethasone-induced bone marrow mesenchymal stem cells, andrographolide reduced the SA-β-gal–positive area as well as p53 and p21 expression and promoted osteogenesis [62]. The expression of osteoblast marker genes *RUNX2*, *COL1A1*, and *ALP* was restored, along with osteocalcin levels. These cellular effects are translated into attenuation of bone loss in vivo, as andrographolide administration (50 mg/kg) increased femoral bone mass and trabecular number. Activation of the PI3K/AKT pathway was identified as a key mechanism underlying these protective effects, and the PI3K inhibitor LY294002 abolished the anti-senescence activity of andrographolide and bone formation [62].

### 2.4. Triterpenoids

#### 2.4.1. Ginsenoside Rg1

Ginsenoside Rg1 (Rg1), a major dammarane-type triterpenoid saponin and active constituent of *Panax ginseng*, is one of the most frequently investigated terpenoids in phytomedical studies. Numerous review articles have summarized the biological activities of Rg1, highlighting its role in cardiovascular diseases [118], neurodegenerative disorders [119,120], ischemic stroke [121], diabetes [122], liver injury [123], and inflammatory diseases [124,125]. Indeed, the anti-aging effects of Rg1 have also been investigated [126], with growing attention focused on its capacity to modulate cellular senescence. The anti-senescence activities of Rg1 and its associated effects on age-related pathologies are outlined in Table 1.

Hematopoietic stem cell (HSC) aging, characterized by diminished self-renewal and skewed lymphoid differentiation, disrupts hematopoietic homeostasis and impairs immune surveillance, thereby driving the onset of age-related myeloproliferative and malignant pathologies [127]. Rg1 consistently improved pathological hematopoietic deterioration by attenuating cellular senescence. Rg1 reduced p16 and p21 expression and recovered the stem cell antigen-1 positive (Sca-1^+^) hematopoietic stem/progenitor cell (HSC/HPC) compartment, implying that Rg1 rejuvenates the pluripotency and self-renewal capacity of HSCs [128]. Moreover, Rg1 restored the mRNA expression of *Cxcl12*, *Kitl*, and *Vcam1*, which play pivotal roles in supporting HSC/HPC survival and differentiation. Conversely, Rg1 dampened the levels of *Selp*, *Nupr1*, and *Mt1*, which are recognized as markers of hematopoietic stem and progenitor cell senescence [63,129]. Mechanistically, transcriptomic analysis showed that TLR2 is a putative upstream target of Rg1. Rg1 reduced D-galactose-induced TLR2 protein expression, and molecular docking predicted an interaction between Rg1 and TLR2 involving the F266 and F284 residues. Consistent with the inhibition of TLR2 signaling, Rg1 suppressed MyD88 expression and decreased IκBα and NF-κB p65 phosphorylation, thereby reducing inflammatory signaling in senescent mesenchymal stromal cells [63].

The deterioration of MSCs through cellular senescence compromises their self-renewal, multilineage differentiation, and immunomodulatory functions, fundamentally undermining both tissue homeostasis and the efficacy of clinical regenerative applications [130]. Rg1 not only restored osteogenic and chondrogenic differentiation capacity but also enhanced the clonogenicity, proliferation, and antioxidant defense of MSCs [64,65]. By modulating GSK-3β phosphorylation, Rg1 prevented excessive WNT/β-catenin activity, which in turn lowered the downstream target T-cell factor/lymphoid enhancer factor (TCF/LEF) and rescued differentiation competence [64]. Moreover, Rg1 activated Nrf2 and its downstream targets, such as HO-1, GCLC, GCLM, and NQO1 [65]. These beneficial changes were attributed to the inhibitory effects of MSC senescence, which diminished p53, p21, and p16 levels and γ-H2AX signals [65,66,131].

Rg1 also exerts protective effects against neural stem cell (NSC) senescence. Chen et al. demonstrated that Rg1 ameliorates cognitive decline, motor dysfunction, and structural deterioration of the hippocampus [67]. Furthermore, Rg1 alleviated brain atrophy and improved the mitochondrial ultrastructure in hippocampal neurons [68]. D-galactose exposure increased the expression of p53, p16, p21, and RB, thereby inducing cell cycle arrest. Rg1 reversed these changes and potentiated the SIRT1/Nrf2/BDNF axis, which is required to restore mitochondrial energy metabolism and diminish ROS levels in NSCs [68]. Moreover, Rg1 suppressed AKT/mTOR phosphorylation and the expression of SASP-related genes [67], suggesting that Rg1 is a promising therapeutic strategy for cognitive health during aging.

In addition, the administration of Rg1 holds medicinal promise for mitigating pulmonary fibrosis, primarily by suppressing cellular senescence [69]. In paraquat-induced C57BL/6 mice, Rg1 reduced the lung wet-to-dry ratio, hydroxyproline content, and collagen deposition. These improvements were driven by reduced p16 and p21 expression, as well as lower levels of SASP factors, including *Tnf*, *Il1b*, *Il6*, *Mmp3*, *Mmp9*, and *Mmp13*. Functionally, Rg1 restored autophagic activity, characterized by the upregulation of the LC3B-II/I ratio and ATG12 and a decrease in p62 [69]. Moreover, Ding et al. revealed that Rg1 acts by inhibiting the cGAS/STING pathway in lung tissue, resulting in diminished chronic inflammation and expression of p16 and p21, which ultimately alleviates pulmonary hypertension [70].

Furthermore, Rg1 attenuates age-related functional impairment in the kidneys. Treatment with Rg1 in senescence-accelerated mouse prone 8 (SAMP8) mice notably restored glomerular pathology, as evidenced by reduced blood urea nitrogen, serum creatinine, and glomerular fibrosis markers, such as collagen IV and TGF-β1 [71]. Similarly, Rg1 ameliorated kidney injury and fibrosis while reducing SA-β-gal, p53, and p21 levels [72]. At the molecular level, Rg1 inhibited the NLRP3 inflammasome, leading to decreased IL-1β signaling [71]. Furthermore, network pharmacology identified caspase-1 as a candidate target, and experiments showed that Rg1 decreased caspase-1, ROS, malondialdehyde (MDA), and TNF-α levels in D-galactose-induced mouse kidneys [72]. Attenuation of cellular senescence also contributes to the improvement of age-related fatty liver disease [73]. By suppressing FOXO1 phosphorylation, Rg1 sustained FOXO1 activity, which in turn elevated superoxide dismutase and catalase activities while lowering SASP-associated cytokine levels, specifically IL-1β, IL-6, and CCL2 [73].

**Figure 2:**
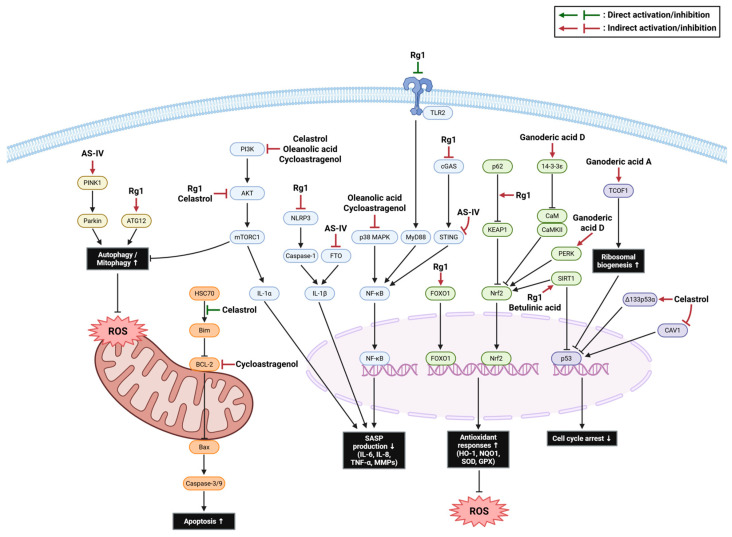
Major schematic pathways of senotherapeutic triterpenoids. Triterpenoids exert senolytic and senomorphic effects through multiple signaling pathways. In the orange-labeled senolytic pathway, celastrol directly binds to HSC70 and disrupts the interaction between HSC70 and Bim [81], whereas cycloastragenol inhibits BCL-2 [90]. These effects activate Bax and caspase 3/9 and promote apoptosis in SnCs. In the yellow-labeled autophagy and mitophagy pathways, astragaloside IV regulates PINK1 and Parkin [75], while Rg1 regulates ATG12 [69]. Celastrol, oleanolic acid, cycloastragenol, and Rg1 also modulate PI3K/AKT/mTORC1 signaling [67,85,90,94]. In the blue-labeled anti-inflammatory pathways, Rg1 regulates TLR2/MyD88 [63], cGAS/STING [70], and NLRP3 [71], while astragaloside IV regulates STING and FTO [79,80]. Oleanolic acid and cycloastragenol further modulate p38 MAPK and NF-κB signaling [90,95]. In the green-labeled antioxidant pathways, Rg1 regulates Nrf2 [65] and FOXO1 [73], ganoderic acid D regulates 14-3-3ε, CaM/CaMKII, and PERK [88,89], and betulinic acid promotes SIRT1 signaling [97]. In the purple-labeled senescence regulatory pathways, ganoderic acid A regulates TCOF1-dependent ribosomal biogenesis [86], while celastrol modulates Δ133p53α [84] and caveolin-1 (CAV1) [82] to reduce cell cycle arrest. Green arrows indicate direct binding. Red arrows indicate indirect modulation. Rg1: ginsenoside Rg1; AS-IV: astragaloside IV. Created in BioRender. Lee, S. (2026) https://BioRender.com/ee0hrte (accessed on 19 July 2026).

Collectively, the available evidence highlights ginsenoside Rg1 as a potent senomorphic agent that counteracts systemic aging by suppressing canonical senescence markers and coordinating multi-target signaling pathways (Figure 2). In hematopoietic and mesenchymal stem cell models, Rg1 primarily restored self-renewal and differentiation, whereas in neural stem cell models it improved mitochondrial function and cognitive phenotypes. In lung, kidney, and liver models, its effects were characterized by the attenuation of fibrotic and inflammatory responses. These model-specific outcomes likely reflect heterogeneity in senescent cell lineage, senescence-inducing stress, and tissue microenvironment.

#### 2.4.2. Astragaloside IV

Astragaloside IV is a cycloartane-type triterpenoid saponin obtained from *Astragalus membranaceus*, a traditional medicinal herb widely used in East Asia. It has been reported to exhibit diverse therapeutic potential, including neuroprotective [132,133,134], cardioprotective [135,136,137], and anti-fibrotic activities [138]. Studies have demonstrated that astragaloside IV effectively attenuates cellular senescence in various cell types.

In neuronal models, astragaloside IV alleviates senescence by enhancing mitophagy and reducing oxidative stress [75,139]. Using both replicative senescent and MPP^+^/LPS-induced premature astrocytes, astragaloside IV significantly promoted mitophagy, as indicated by the recovery of LC3-II, PINK1, and Parkin, depletion of p62 and TOM20, and suppression of mitochondrial ROS accumulation [75]. A parallel role of mitophagy quality control has also been observed in bleomycin-induced vascular smooth muscle cells (VSMCs) [76]. Additionally, in amyloid-beta (Aβ)-induced human astrocytes, astragaloside IV inhibited the chaperone protein HSP90AA1 [78], which is linked to the apoptosis resistance of SnCs. Because HSP90 inhibition destabilizes pro-survival client proteins such as phosphorylated AKT and suppresses PI3K/AKT-dependent anti-apoptotic signaling, targeting HSP90AA1 may represent a relevant mechanism that sensitizes SnCs to apoptosis [20].

Consistent with these in vitro results, astragaloside IV has shown promising therapeutic outcomes in several in vivo age-related pathologies, including pulmonary fibrosis [140], myocardial fibrosis [141], osteoporosis [79], age-related macular degeneration [80], cognitive impairment [77], and Parkinson’s disease [75]. Astragaloside IV attenuated pulmonary and myocardial fibrotic injury, as indicated by improved histopathology, reduced collagen deposition, lower levels of fibrosis-associated markers (collagen I/III and α-SMA), and SASP expression, including IL-1β, IL-6, TNF-α, and TGF-β1 [140,141]. Integrated transcriptomic and proteomic profiling combined with KEGG enrichment analysis revealed that astragaloside IV targets pathways involving cellular senescence and p53 signaling [141], suggesting that the modulation of senescence and SASP signaling by astragaloside IV may represent a shared mechanism underlying its therapeutic potential in age-related fibrotic diseases [142].

Furthermore, astragaloside IV mitigated D-galactose-induced bone deterioration in C57BL/6J mice, improving trabecular microarchitecture and reducing senescent phenotypes in bone marrow mesenchymal stem cells (BMSCs) [79]. In particular, macrophage polarization shifted away from a pro-inflammatory M1-like state toward an anti-inflammatory M2-like phenotype. The suppression of the STING/NF-κB axis was identified as the underlying signaling pathway driving the rescue of macrophage senescence and, in turn, promoting the osteogenic differentiation of BMSCs [79,143].

Astragaloside IV also ameliorated NaIO_3_-induced retinal pathology in mice by improving retinal structure and thickness while decreasing p53 expression and γ-H2AX signal levels [80]. Mechanistically, astragaloside IV directly interacts with the fat mass and obesity-associated (FTO) protein, which is required for m6A modification of *Il1β* mRNA to reduce its stability [80].

Taken together, these findings indicate that astragaloside IV exerts senotherapeutic and tissue-protective effects for several age-related disorders by suppressing canonical senescence and inflammatory phenotypes while restoring mitochondrial quality control and modulating key signaling nodes, including PINK1/Parkins, STING/NF-κB, and FTO-dependent post-transcriptional regulation (Figure 2).

#### 2.4.3. Celastrol

Celastrol is a quinone methide pentacyclic triterpenoid originally isolated from *Tripterygium wilfordii* and other Celastraceae species, which are abundantly distributed across China and Southeast Asia. Celastrol exerts diverse bioactivities, including anticancer [144], antimicrobial [145], anti-inflammatory, metabolic regulatory, and neuroprotective effects [145].

Celastrol was identified as a novel senolytic compound that preferentially eliminates SnCs in stress-induced NIH-3T3 and human umbilical vein endothelial cells (HUVECs), as well as replicative senescent mouse embryonic fibroblasts (MEFs) [81]. Celastrol stabilized the pro-apoptotic protein Bim and disrupted the HSC70–Bim interaction, which normally facilitates Bim degradation through CHIP-dependent ubiquitination. Moreover, celastrol modestly extended lifespan in *Drosophila* and attenuated bleomycin-induced lung fibrosis and CCl_4_-induced liver fibrosis in C57BL/6 mice, along with increased apoptosis of p16-positive cells [81].

Celastrol also exerts anti-senescent effects in renal cell carcinoma [82] and tubular epithelial cells [83]. Celastrol administration in clear cell renal cell carcinoma (ccRCC) effectively alleviated doxorubicin-induced senescence by downregulating caveolin-1, which promotes tumor senescence via the p53/p21^Waf1/Cip1^ pathway [146]. This pharmacological intervention neutralized the deleterious paracrine effects of SnCs, including enhanced migration, invasion, epithelial–mesenchymal transition, and stemness [82]. Moreover, the protective action in renal tubular epithelial cells was associated with the suppression of AKT1 and NF-κB phosphorylation, suggesting that celastrol interrupts senescence-linked inflammatory responses [83].

Moreover, in a quantitative high-throughput screening assay using doxycycline-inducible eGFP-tagged Δ133p53α, cellular GFP fluorescence intensity was measured to identify compounds that increase Δ133p53α protein levels. As Δ133p53α is known to antagonize full-length p53 and block its function [147,148], it strongly suppresses the expression of p21 and SASP factors. Celastrol was selected based on its potency, absence of cytotoxicity, and reproducibility across Δ133p53α reporter systems. Subsequently, the effect of celastrol was validated to increase endogenous Δ133p53α levels in primary human astrocytes and MRC-5 fibroblasts, with a concomitant reduction in IL-6 secretion and SA-β-gal positivity [84]. Additionally, celastrol inhibited the PI3K/AKT/mTOR/p70S6K signaling axis, triggering an autophagic response that restored the cell cycle in rat VSMCs [85].

Altogether, these studies indicate that celastrol elicits heterogeneous senotherapeutic responses across experimental models. It promotes Bim-dependent apoptosis in senescent fibroblasts and endothelial cells, whereas in renal, astrocytic, and vascular models it mainly attenuates senescence-associated signaling through CAV1/p53/p21, AKT/NF-κB, Δ133p53α, or autophagy (Figure 2).

#### 2.4.4. Ganoderic Acid A and D

Ganoderic acids are a class of tetracyclic triterpenoids predominantly isolated from the medicinal fungus *Ganoderma lucidum*, a traditional herbal medicine widely used in East Asia to promote health and longevity. Numerous ganoderic acid derivatives have been identified, and these compounds exhibit diverse biological activities, including antioxidant, immunomodulatory, and anticancer activities [149,150].

Studies have elucidated their roles in modulating cellular senescence and aging-related pathways. Using a high-content screening platform, Chen et al. screened 805 natural products in replicative senescent IMR90 cells using multiparametric senescence readouts, including SA-β-gal positivity, cell number, nuclear area, cell area, and morphology [86]. Subsequent validation across diverse models, including replication-, oxidative stress-, and genotoxicity-induced senescent HUVECs, identified ganoderic acid A as a lead senotherapeutic candidate. Notably, it consistently reduced the number of SA-β-gal-positive cells while demonstrating a wide effective concentration range and low cytotoxicity in senescent MEFs and L02 cells [86]. Ganoderic acid A significantly extended the healthspan of X-ray-irradiated prematurely aged, naturally aged, and Western diet-induced obese C57BL/6J mice. Systemic administration of ganoderic acid A mitigated the accumulation of SnCs and curtailed multi-organ physiological deterioration (creatine kinase-MB, creatinine, ALT, and AST), while notably enhancing physical capacity and preserving metabolic homeostasis without triggering abnormal cellular proliferation [86].

Mechanistic analysis revealed that ganoderic acid A directly binds to TCOF1, a nucleolar protein that regulates ribosomal RNA transcription and its biogenesis. Cellular senescence is frequently accompanied by nucleolar stress and impaired ribosomal production [151], leading to translational dysfunction and activation of stress signaling pathways by impairing MDM2-mediated ubiquitination of p53 [152,153]. By stabilizing the phosphorylated form of TCOF1, ganoderic acid A effectively maintained ribosomal protein production and global translation capacity, as evidenced by the restored O-propargyl puromycin (OPP) incorporation in SnCs. Furthermore, siRNA-mediated knockdown of TCOF1 completely abrogated the recovery of ribosomal function and its subsequent anti-senescent effects, confirming that the preservation of TCOF1-dependent ribosome biogenesis is indispensable for the senotherapeutic action of ganoderic acid A [86].

Also, treatment with ganoderic acid A in Alzheimer’s disease models significantly reduced senescence phenotypes by activating autophagic pathways, including ATG5 and Beclin-1. Specifically, PADI4 knockdown abrogated the protective effects of ganoderic acid A, indicating that PADI4 may play a significant role in mediating ganoderic acid A-induced autophagy activation [87].

In hydrogen peroxide–treated human amniotic mesenchymal stem cells (hAMSCs), ganoderic acid D prevented cell cycle arrest while enhancing antioxidant responses, such as HO-1 and NQO1 [88,89]. Ganoderic acid D activated Nrf2-dependent antioxidant signaling through PERK phosphorylation, promoting Nrf2 nuclear translocation and PRDX3 induction [89]. In parallel, ganoderic acid D stabilized 14-3-3ε interactions with CaM or phosphorylated CaMKII. This regulation of the Ca^2+^-sensing CaM/CaMKII module upstream of Nrf2 likely facilitated nuclear translocation of Nrf2 and restoration of calcium/redox homeostasis [88]. These molecular events translated to enhanced systemic antioxidative defense and improved physical endurance in D-galactose-induced in vivo models [88].

Additionally, *G. lucidum* demonstrates a clear capacity to mitigate cellular senescence when administered as a crude extract. Abdelmoaty et al. reported that *G. lucidum* extract, rich in ganodermanontriol, ganodermanondiol, ganoderiol A/B, and ganoderal A, possesses senolytic activity against adriamycin-induced hepatocellular carcinoma cells [154]. This complex triggered apoptosis via caspase-dependent and mitochondrial pathways, which is further supported by the downregulation of anti-apoptotic BCL-2 family members and the PI3K/AKT axis [154]. Also, *G. lucidum* extract induced the expression of antioxidant defense genes, including HMOX1, GCLM, and NQO1, to alleviate senescence in human dermal fibroblasts (HDFs) [155].

In summary, these studies suggest that ganoderic acids, particularly ganoderic acid A and D, confer substantial senescence-modulating effects by improving age-related physiological impairment and reducing multiple senescence markers by preserving nucleolar and ribosomal function, promoting autophagic flux, and strengthening Nrf2-driven cytoprotective antioxidant signaling (Figure 2). Furthermore, *G. lucidum* extracts exhibit broad anti-senescence potential across diverse cellular contexts, including both malignant and non-malignant proliferative cells.

#### 2.4.5. Cycloastragenol

Cycloastragenol, a bioactive triterpene aglycone derived from *Astragalus*, has been shown to possess versatile properties, including telomerase activation [156], proteasome activity [157], circadian rhythm regulation [158], and anti-inflammatory responses [159]. Zhang et al. investigated the senolytic activity of cycloastragenol in etoposide-induced IMR90 fibroblasts. This study revealed the capacity of cycloastragenol to selectively eliminate SnCs by suppressing the PI3K/AKT/mTOR and anti-apoptotic BCL-2 family signaling pathways [90]. Additionally, cycloastragenol attenuated microglial senescence and cognitive deficits in 5xFAD mice by targeting PDE4B to enhance microglial phagocytosis and activate the CREB/BDNF signaling pathway [91]. In contrast to inducing apoptosis in SnCs, cycloastragenol preserved nucleus pulposus cell proliferation by reducing p16, cleaved caspase-3, and BAX [92]. Taken together, cycloastragenol exerts distinct senotherapeutic effects across different models, including direct senolysis in senescent IMR90 cells, attenuation of microglial senescence in 5xFAD mice, and preservation of nucleus pulposus cell proliferation under high-glucose conditions (Figure 2).

#### 2.4.6. Oleanolic Acid

Oleanolic acid is a naturally occurring pentacyclic triterpenoid that is abundant in olive leaves (*Olea europaea* L.). Oleanolic acid exhibits broad pharmacological efficacy, including antidiabetic, anticancer, antihypertensive, hepatoprotective, and neuroprotective effects [160,161]. In an intestinal injury model induced by 5-fluorouracil, oleanolic acid attenuated senescence in HUVECs and NCM460 cells, reduced SASP gene expression (*IL1B*, *IL6*, *IL8*, *IFNG*, and *TNF*), and ameliorated diarrhea, colonic shortening, and crypt disruption in BALB/c mice [93]. Functionally, oleanolic acid suppressed mTOR signaling, p-p65, and p-p38 [93], consistent with a report that oleanolic acid blunted senescence in HDFs and MEFs by targeting the IGF-1-mediated PI3K/AKT/mTOR pathway [94]. In addition, it was demonstrated that inhibiting the JNK/MAPK pathway to suppress senescence is another mechanism of action of oleanolic acid by treating a zebrafish atherosclerosis model with oleanolic acid nanoparticles [95]. Collectively, these studies suggest that oleanolic acid functions as an anti-senescence compound by coordinating the inhibition of the PI3K/AKT/mTOR, p38/NF-κB, and JNK/MAPK signaling pathways, respectively (Figure 2).

#### 2.4.7. Betulinic Acid

Betulinic acid, a lupane-type pentacyclic triterpenoid enriched in the bark of *Betula* and *Ziziphus* species, is well known for its antioxidant, anti-inflammatory, and anticancer activities [162,163,164]. Following reports of lifespan extension by betulinic acid in *S. cerevisiae* [165], *C. elegans* [166], and *D. melanogaster* [167], its potential senotherapeutic properties have also been explored. Odama et al. demonstrated that betulinic acid effectively mitigates replicative senescence in high-passage fibroblasts. Transcriptomic analysis revealed that betulinic acid suppresses type I interferon programs, including IFI6 and MX1, by downregulating IRF9 [96]. Moreover, a network pharmacology study suggested that betulinic acid may involve a broader multi-target mechanism, particularly p53 and SIRT1, as important interacting nodes. Indeed, it was experimentally confirmed that betulinic acid modulates the SIRT1-p53 signaling axis and promotes HK-2 cell proliferation, accompanied by downregulation of the expression of the inflammatory markers TGF-β and IL-6 [97]. Overall, these findings indicate that betulinic acid attenuates cellular senescence by modulating a wide range of inflammatory factors.

## 3. Conclusions

The aging population has increased rapidly on a global scale, posing a major socioeconomic burden. Life expectancy continues to increase, but the healthspan fails to keep pace with the lifespan [168]. This gap highlights the need for therapeutics that promote healthy aging. Cellular senescence is now recognized as an essential contributor to the fundamental aging process and etiology of age-related diseases [169]. Accordingly, targeting cellular senescence has emerged as a promising anti-aging strategy, and the development of safe and effective senotherapeutics has become increasingly important. Natural compounds, in particular, have garnered increasing attention for their potential to alleviate cellular aging. Although their potency may be lower than that of synthetic compounds, they are generally considered safe, and their lower toxicity makes them more favorable for clinical use. Among the various phytocompounds, this review focuses on terpenoids and comprehensively summarizes their senescence-modulating roles at the cellular, tissue, and organismal levels.

Plant-derived terpenoids are mainly obtained from aromatic and essential-oil-rich plants and from perennial medicinal herbs, shrubs, and trees distributed across temperate East Asia, tropical and subtropical Asia, the Mediterranean region, and temperate Eurasia. Numerous studies have shown that terpenoids exert senotherapeutic effects and mitigate the detrimental effects of aging. Notably, these compounds directly eliminate SnCs by apoptosis or ferroptosis and suppress the senescence-associated secretory phenotype (SASP), which fuels age-related degeneration [30]. These effects are mediated by multiple regulatory pathways rather than a single response. Specifically, terpenoids modulate key signaling pathways associated with cellular senescence, including inflammatory pathways such as NF-kB, JAK/STAT, PI3K/AKT/mTOR, and p38 MAPK signaling pathways. In addition, certain terpenoids regulate redox homeostasis through Nrf2 activation and modulate core cellular processes, such as mitochondrial function and autophagy. Taken together, terpenoids not only reduce the accumulation of SnCs but also modulate interconnected senescence-associated networks, thereby attenuating the initiation and progression of senescence.

Monoterpenoids and sesquiterpenoids predominantly exert senotherapeutic effects through SASP modulation (Figure 1). While thymol promotes senescent cell apoptosis and dihydroartemisinin induces ferroptosis-dependent senolysis through AMPK activation and autophagic ferritin degradation, most other compounds function as senomorphic agents. Limonene suppresses NF-κB inflammatory signaling, whereas thymol and carvacrol attenuate oxidative stress and inhibit JAK/STAT3-driven senescence pathways. β-Caryophyllene promotes longevity through SIRT1 induction, and handelin suppresses p38 MAPK activation and enhances AKT/mTORC1 signaling. Diterpenoids provide broader protection against age-related dysfunction. Oridonin displays senolytic activity via GSTK1 inhibition and p38-mediated apoptosis, whereas ginkgolide B and andrographolide alleviate frailty, age-associated muscle decline, and bone loss by suppressing SASP-associated inflammation. Tanshinone IIA further protects against vascular aging by inhibiting NF-kB/NLRP3 signaling. Meanwhile, triterpenoids provide multifaceted senotherapeutic regulation, acting through both senolytic and senomorphic mechanisms (Figure 2). In the senolytic context, celastrol and cycloastragenol exhibit senolytic activity by enhancing apoptotic susceptibility through Bim stabilization or inhibition of PI3K/AKT/mTOR and BCL-2 family signaling. On the other hand, ginsenoside Rg1, astragaloside IV, ganoderic acids, oleanolic acid, and betulinic acid mainly act as senomorphic agents. Rg1 and oleanolic acid suppress NF-κB, SASP, and mTOR-linked inflammatory pathways. Astragaloside IV restores PINK1/Parkin-mediated mitophagy and regulates STING/NF-κB signaling. Ganoderic acids reinforce ribosomal homeostasis, autophagy, and Nrf2-dependent antioxidant defense, while betulinic acid attenuates interferon-driven inflammation and modulates the SIRT1–p53 axis.

The aforementioned ability of terpenoids to improve cellular senescence at multiple levels highlights their potential as anti-senescence agents. However, several limitations remain. Most current studies are based on phenotypic observations and describe pathway alteration without a complete understanding of molecular targets or underlying mechanisms. Indeed, only a limited number of compounds have defined direct molecular targets, including oridonin (GSTK1), celastrol (HSC70), and ganoderic acid A (TCOF1). Target identification of the remaining compounds should be prioritized in future studies. In addition, much of the existing evidence is derived from preclinical studies and therefore may not fully capture the complexity of in vivo aging. Although a few candidate terpenoids and their derivative formulations have entered clinical evaluation for aging-associated phenotypes and dysfunction (NCT05578443, NCT05647473, NCT05598359), most of these trials are still in their nascent stages or remain ongoing, leaving publicly available efficacy data highly sparse. Further validation using diverse animal models and well-designed clinical trials should be conducted to confirm their effects on senescence.

Another important consideration is the heterogeneity of cellular senescence, posing a challenge to the generalization of existing findings. Senescent phenotypes and responses vary across cellular and tissue environments, and whether comparable effects occur in different models remains unclear. This variability necessitates context-dependent validation and careful optimization. Furthermore, a deeper understanding of pharmacokinetics and toxicology, along with ADME assessment, is required to facilitate the development of terpenoid-based senotherapeutics. While the terpenoids discussed in this review are naturally derived and generally considered to have favorable safety profiles, their long-term safety under aged conditions should be addressed.

In conclusion, this review provides a comprehensive analysis of the senotherapeutic potential of terpenoids, which have been relatively underexplored. Terpenoids combat cellular aging and suppress senescent phenotypes, thereby emerging as promising candidates for promoting healthy aging. Although terpenoid-based senothereapeutics remain at an early stage of development, accumulating findings support their promising senotherapeutic activities. We believe that terpenoids could possibly serve as a novel class of drugs for age-related diseases.

## Figures and Tables

**Table 1:** Senotherapeutic activity of terpenoids.

Compound	Senescence and Aging Models	Senescence Markers	Effects and Mechanisms	Ref.
*Monoterpenoids*				
Limonene	UVB-irradiated HaCaT cells	p53 ↓ MMP2 ↓	Activation of Nrf2 via JNK/SAPK and AKT phosphorylation	[39]
	D-galactose-induced Swiss albino mice	IL-6, IL-1β, TNF-α ↓	Attenuation of epidermal thinning Inhibition of NF-kB activation	[40]
Thymol	H_2_O_2_-induced MSCs	SA-β-Gal ↓ p53, p16, RB2 ↓ pSRC/SRC ↓	Induction of caspase-independent apoptosis via AIF translocation	[35]
	SAMP8 mice	Epigenetic age ↓	Suppression of NF-kB and IL-6/JAK/STAT3 signaling	[41]
	tert-Butyl hydroperoxide-treated KGN cells	SA-β-Gal ↓ p53, p21, p16 ↓	Inhibition of JAK1/STAT3 signaling	[42]
Carvacrol	H_2_O_2_-induced MSCs	SA-β-Gal ↓ γ-H2AX ↓ p53, p21, p16, p27, RB2 ↓	Induction of caspase-dependent apoptosis	[35]
Eugenol	H_2_O_2_-induced MSCs	SA-β-Gal ↓ γ-H2AX ↓ p53, p21, p16, p27, RB2 ↓ pSRC/SRC ↓	Induction of caspase-independent apoptosis via AIF translocation	[35]
*Sesquiterpenoids*				
Dihydroartemisinin	H_2_O_2_-induced NIH3T3 cells	SA-β-Gal ↓ p53, p21, p16 ↓ PTGS2 ↑	Initiation of autophagy-dependent ferroptosis via the AMPK/mTORC1 pathway	[43]
	R848-treated myeloid-derived suppressor cells	SA-β-Gal ↓ p53, p21, p16 ↓ IL-1β, IL-6, IL-8, TNF-α ↓	Activation of the Nrf2/HO-1 pathway Protective effect against systemic lupus erythematosus manifestations	[44]
	Pristane-induced lupus BALB/c mice			
	TNF-α-induced nucleus pulposus cells	SA-β-Gal ↓ p21, p16 ↓ IL-1β, IL-6, IL-8, MMP3, MMP9 ↓ IL-10 ↑	Inhibition of the NF-kB pathway induced by TNF-α Suppression of PI3K/AKT signaling	[45]
	Puncture-induced rats	p16 ↓ IL-6, MMP13 ↓	Attenuation of intervertebral disc degeneration onset and progression	
	Renal ischemia/reperfusion injury surgical model ICR mice	SA-β-Gal ↓ γ-H2AX ↓ p21, p16 ↓ CDK4, Cyclin D1 ↑ *Il6*, *Il8*, *Tnf*, *Cxcl1* ↓	Induction of autophagic flux in renal tubular epithelial cells	[46]
	Unilateral ureteral obstruction model ICR mice			
	Blood-induced murine primary chondrocytes	SA-β-Gal ↓ p53, p21, p16 ↓ MMP3, MMP13 ↓	Activation of the Nrf2 signaling pathway in cartilage by enhancing Keap1 ubiquitination and Nrf2 translocation	[47]
	F8^-/-^ hemarthrosis C57BL/6J mice	p16 ↓ MMP13 ↓	Alleviation of cartilage hemarthrosis through Nrf2/Keap1 signaling Attenuation of subchondral bone loss	
Handelin	UVB-induced HaCaT cells	p38, ERK1/2 ↓ MMP2, MMP9 ↓	Inhibition of p38 MAPK phosphorylation	[48]
	TNF-α induced C2C12 myotube	*Il6*, *Il1b*, *Tnf*, *Cxcl1* ↓ p65 ↓	Activation of AKT-mTORC1 signaling Suppression of NF-kB pathway	[49]
	LPS-induced C57BL/6 mice		Activation of IGF-1/AKT signaling Alleviation of skeletal muscle loss and atrophy through Hsp70 activation	
β-caryophyllene	*C. elegans*	Lipofuscin ↓	Longevity promotion mediated by *skn-1* and *sir-2.1*	[50]
	Replicative senescent HUVECs	p16^INK4a^ ↓ *IL1B*, *IL6*, *TNF* ↓ miR-146a, miR-21 ↓	Induction of SIRT1 expression Mitigation of inflammaging	[51]
	LPS-stimulated THP-1 monocytic cells			
*Diterpenoids*				
Ginkgolide B	H_2_O_2_-induced C2C12 myoblast	SA-β-Gal ↓ γ-H2AX ↓ *Cdkn1a*, *Cdkn2a*, *Trp53* ↓ *Il6*, *Ifn-γ* ↓	Suppression of Runx1 expression in muscle Mitigation of age-related muscle wasting by restoring miR-27b-3p levels	[52]
	Naturally aged C57BL/6 female mice	γ-H2AX ↓ *Cdkn2a*, *Cdkn2d*, *Cdkn1c* ↓ *Il6*, *Ifng* ↓		
	BaCl_2_-induced C57BL/6 mice	*Il1b*, *Tnf*, *Tgfb1* ↓	Reactivation of osteocalcin-GPRC6A signaling Enhancement of muscle regeneration	[53]
Oridonin	Cisplatin-induced A549 cells	SA-β-Gal ↓ p21, p16 ↓	Induction of senolysis via ROS-activated p38 signaling	[54]
	Bleomycin-induced BJ cells	IL-6, IL-8, CCL20 ↓	Downregulation of NF-kB Inactivation of p38 pathway	[55]
	Doxorubicin-induced WI-38, 2BS cells	SA-β-Gal ↓ p53, p21 ↓ *Il1a*, *Il1b*, *Il6*, *Il8* ↓	Inhibition of the AKT-mediated FOXO1 phosphorylation Suppression of NLRP3 inflammasome	[56]
Tanshinone IIA	High glucose-induced HPMCs	SA-β-Gal ↓ p21, p16 ↓	Prevention of cell cycle arrest and telomere shortening	[57]
	H_2_O_2_-induced HUVECs	SA-β-Gal ↓ p21, p16 ↓	Activation of the SIRT1/eNOS axis	[58]
Sodium tanshinone IIA sulfonate	High glucose-treated primary endothelial cells	SA-β-Gal ↓ p21 ↓ IL-1β, IL-18 ↓	Inhibition of the NF-κB pathway and NLRP3 inflammasome activation Attenuation of vascular senescence	[59]
	High glucose-treated VSMCs			
	High glucose-induced endothelial progenitor cells	SA-β-Gal ↓ γ-H2AX ↓ p21 ↓ IL-1β ↓	Inhibition of the NLRP3 inflammasome via RAGE-TXNIP pathway	[60]
Andrographolide	HFD-fed C57BL/6 mice	SA-β-Gal ↓ p53, p21, p16 ↓ *Il6*, *Il1a*, *Il1b*, *Cxcl10* ↓ Mmp9 ↓	Activation of AMPK signaling in the kidney Mitigation of renal fibrosis	[61]
	Palmitic acid-induced HK-2 cells	SA-β-Gal ↓ p53, p16 ↓	Alleviation of mitochondrial damage	
	Dexamethasone-induced BMSCs	SA-β-Gal ↓ p53, p21, p16 ↓	Promotion of osteogenic differentiation via PI3K/AKT activation	[62]
	Dexamethasone-induced C57BL/6 mice	p53, p21, p16 ↓	Attenuation of bone loss in femurs	
*Triterpenoids*				
Ginsenoside Rg1	HSPCs co-cultured with D-galactose-induced BMSCs	SA-β-Gal ↓ p53, p21 ↓ IL-1β, IL-6, TNF-α ↓	Suppression of TLR2/NF-κB signaling via TLR2 receptor binding	[63]
	hBM-MSCs from aged donors	SA-β-Gal ↓ p53, p16 ↓	Suppression of WNT/β-catenin signaling via inhibition of GSK-3β phosphorylation	[64]
	D-galactose-induced BM-MSCs	SA-β-Gal ↓ p53, p21, p16 ↓ γ-H2AX ↓ *Il1b*, *Il6*, *Mmp3*, *Mmp12* ↓	Activation of the Nrf2 pathway via p62- mediated KEAP1 degradation	[65]
	D-galactose-induced C57BL/6J mice		Elevation of Nrf2 nuclear translocation in heart, liver, and lung tissues	
	H_2_O_2_-induced ADSCs	SA-β-Gal ↓ p21, p16 ↓ γ-H2AX ↓ *IL1B*, *IL6*, *TNF* ↓	Activation of the PI3K/AKT signaling pathway to restore stemness	[66]
	D-galactose-induced NSCs	SA-β-Gal ↓ p53, p21, p16, RB ↓	Downregulation of the AKT/mTOR signaling pathway in neural stem cells	[67]
	D-galactose-induced C57BL/6 mice			
	D-galactose-induced NSCs	SA-β-Gal ↓ p53, p21, p16 ↓ *Il1a*, *Il6*, *Tnf*, *Tgfb1*, *Ccl5* ↓	Activation of the SIRT1/Nrf2/BDNF signaling pathway in both primary neural stem cells and hippocampal tissue	[68]
	D-galactose-induced C57BL/6 mice			
	Paraquat-induced MLE-12 cells	SA-β-Gal ↓ p21, p16 ↓ *Il1b*, *Il6*, *Tnf*, *Mmp3*, *Mmp9*, *Mmp13* ↓	Promotion of autophagy via ATG12 induction and activation of caspase-3 in both MLE-12 cells and lung tissue	[69]
	Paraquat-induced C57BL/6 mice			
	Sugen/hypoxia-induced rats	p21, p16 ↓ IL-6 ↓	Inhibition of the cGAS/STING signaling pathway in lung tissue	[70]
	SAMP8 mice	SA-β-Gal ↓ IL-1β, TGF-β1 ↓	Inhibition of NLRP3 inflammasome in renal cortex and glomerular tissue	[71]
	D-galactose-induced C57BL/6J mice	SA-β-Gal ↓ p53, p21 ↓	Inhibition of caspase-1 activity in kidney tissue	[72]
	D-galactose-induced C57BL/6J mice	SA-β-Gal↓ p53, p21↓ IL-1β, IL-6, CCL2 ↓	Inhibition of FOXO1 phosphorylation to induced SOD and catalase production in liver tissue	[73]
	High glucose-induced rat retinal ganglion cells	SA-β-Gal ↓ p53, p21, p16 ↓ Lamin B1 ↑	Restoration of mitochondrial function by promoting PGC-1α transcription via VDR/cAMP/PKA/CREB signaling axis	[74]
Astragaloside IV	Replicative senescent or LPS/MPP^+^-induced primary astrocytes	SA-β-Gal ↓ p16 ↓ Lamin B1 ↑ *Il1a*, *Il1b*, *Il6*, *Cxcl1*, *Mmp3*, *Mmp9* ↓	Restoration of PINK1/Parkin to induce mitophagy in primary astrocytes	[75]
	MPTP-induced mice			
	Bleomycin-induced VSMCs	SA-β-Gal ↓ p21, p16 ↓ DcR2 ↓	Activation of Parkin to induce mitophagy in both VSMCs and aorta tissue	[76]
	D-galactose-induced BALB/c mice			
	UVA radiation-induced PC12 and primary neuronal cells	SA-β-Gal ↓ p21, RB ↓ CDK2 ↑	Activation of the ERK signaling pathway in both primary cortical neurons and cerebral cortex tissue	[77]
	UVA radiation-induced C57BL/6 mice			
	Oligomerized Aβ-induced primary human astrocytes	SA-β-Gal ↓ p53, p21, p16 ↓	Inhibition of HSP90AA1 protein levels to disrupt stabilization of pro-survival molecules	[78]
	H_2_O_2_-induced RAW264.7 cells	SA-β-Gal ↓ p53, p21, p16 ↓ *Il6*, *Tnf* ↓	Inhibition of STING/NF-κB signaling pathway	[79]
	BMSCs treated with senescent macrophage conditioned medium			
	NaIO_3_-induced ARPE-19 cells	SA-β-Gal ↓ p53, p21 ↓ γ-H2AX ↓ *Il1b*, *Il6* ↓	Destabilization of IL-1β mRNA via FTO- mediated m6A modification in both ARPE-19 cells and retinal tissue	[80]
	NaIO_3_-induced C57BL/6J mice			
	Isoproterenol-induced C57BL/6J mice			
Celastrol	H_2_O_2_, Adriamycin, or HG-induced HUVECs and NIH3T3 cells	SA-β-Gal ↓ p21, p16 ↓	Inhibition of HSC70-Bim-CHIP complex to disrupt ubiquitination of pro-apoptotic Bim protein	[81]
	Bleomycin or CCl_4_-induced IPF C57BL/6 mice		Activation of caspase-3 to eliminate SnCs in lung and liver tissues	
	Doxorubicin-induced 786-O and A498 cells	SA-β-Gal ↓ p53, p21, p16 ↓ IL-6, IL-8, CXCL12 ↓	Downregulation of caveolin-1 expression to suppress the p53/p21^Waf1/Cip1^ pathway in clear cell renal cell carcinoma	[82]
	Subcutaneous ccRCC xenograft C57BL/6J mice			
	High glucose-induced HK-2 cells	SA-β-Gal ↓ *CDKN1A*, *CDKN2A* ↓ *IL1B*, *CCL2*, *TNF* ↓	Inhibition of the AKT/NF-κB/TNF-α signaling pathway in both HK-2 cells and kidney tissue	[83]
	Streptozotocin-induced SD rats			
	Replicative senescent human primary astrocytes and MRC-5 cells	SA-β-Gal ↓ IL-6 ↓	Antagonization of p53 by elevating Δ133p53α protein levels	[84]
	Angiotensin II-induced primary rat VSMCs	SA-β-Gal ↓ p53, p21 ↓	Induction of autophagy through inhibition of the PI3K/AKT/mTOR signaling pathway	[85]
Ganoderic acid A	Replicative senescent IMR90 cells	SA-β-Gal ↓ p53, p21, p16, Cyclin D1 ↓ γ-H2AX ↓ IL-6 ↓	Maintenance of ribosomal homeostasis and biogenesis by stabilizing TCOF1 phosphorylation via direct binding	[86]
	H_2_O_2_, Etoposide, or CX-5461-induced HUVECs			
	Naturally aged or IR-induced C57BL/6J mice		Upregulation of the ribosomal pathway in heart and lung tissues	
	Aβ25-35-induced HT22 cells	SA-β-Gal ↓ p21, p16 ↓ HMGA1 ↓	Upregulation of PADI4 to suppress AKT/mTOR signaling and enhance autophagic flux	[87]
Ganoderic acid D	H_2_O_2_-induced hAMSCs	SA-β-Gal ↓ p21, p16 ↓	Modulation of CaM/CaMKII/NRF2 signaling through 14-3-3ε interaction	[88]
	D-galactose-induced ICR mice		Promotion of Nrf2 nuclear translocation by elevating 14-3-3ε expression in bone marrow stem cells	
	H_2_O_2_-induced hAMSCs	SA-β-Gal ↓ p21, p16 ↓	Activation of the PERK/Nrf2 signaling pathway	[89]
Cycloastragenol	Etoposide-induced IMR90 cells	SA-β-Gal ↓ p53, p21, p16 ↓ *Il6*, *Cxcl5*, *Cxcl10* ↓	Inhibition of BCL-2 family, PI3K/AKT/mTOR, p38 MAPK/NF-κB, and JAK/STAT3 signaling pathways in both IMR90 cells and inguinal adipose tissue	[90]
	Irradiation-induced C57BL/6 mice			
	5xFAD-induced C57BL/6J mice	SA-β-Gal ↓ *Cdkn1a*, *Cdkn2a* ↓	Inhibition of PDE4B to activate CREB/BDNF signaling and promote microglial phagocytosis of SnCs	[91]
	High glucose-induced rat NPCs	SA-β-Gal ↓ p16 ↓	Upregulation of TERT expression	[92]
Oleanolic acid	5-Fluorouracil-induced HUVECs and NCM460 cells	SA-β-Gal ↓ *TP53*, *CDKN1A* ↓ p16 ↓ *IL1B*, *IL6*, *IL8*, *IFNG*, *TNF* ↓	Inhibition of mTOR signaling pathway in both NCM460 cells and colon tissue	[93]
	5-Fluorouracil-induced BALB/c mice			
	Bleomycin-induced HDFs and MEFs	SA-β-Gal ↓ p16 ↓ IL-1β, IL-6, IL-8 ↓	Inhibition of IGF-1 expression and PI3K/AKT/mTOR signaling pathway	[94]
	oxLDL-induced fli1a::EGFP^+^ zebrafish	SA-β-Gal ↓	Inhibition of JNK/MAPK pathway in zebrafish endothelial cells	[95]
Betulinic acid	Replicative senescent or etoposide-induced HDFs	SA-β-Gal ↓ *CDKN1A* ↓	Suppression of IFN-inducible genes through downregulating IRF9 expression	[96]
	D-galactose-induced HK-2 cells	SA-β-Gal ↓ p53, p21 ↓ IL-6, TGF-β1 ↓	Inhibition of BCL-2 family, PI3K/AKT/mTOR, p38 MAPK/NF-κB, and JAK/STAT3 signaling pathways in both IMR90 cells and inguinal adipose tissue	[97]

↓ indicates a decrease in expression and ↑ indicates an increase in expression.

## Data Availability

No new data were created or analyzed in this study. Data sharing is not applicable to this article.

## Abbreviations

The following abbreviations are used in this manuscript:

53BP1	p53-Binding Protein 1
ADME	Absorption, Distribution, Metabolism, Excretion
AIF	Apoptosis-Inducing Factor
AKT	Protein Kinase B
ALT	Alanine aminotransferase
AMPK	AMP-activated Protein Kinase
AST	Aspartate Transaminase
ATG12	Autophagy Related 12
BAX	Bcl-2-Associated X Protein
BCL2	B-cell Lymphoma 2
BDNF	Brain-Derived Neurotrophic Factor
cAMP	Cyclic Adenosine Monophosphate
CCL2	C-C motif Ligand 2
cGAS	Cyclic GMP-AMP Synthase
CHIP	Carboxyl terminus of HSC70-Interacting Protein
CREB	Cyclic AMP-Responsive Element Binding Protein
eNOS	Endothelial Nitric Oxide Synthase
ERK	Extracellular Signal-Regulated Kinase
FOXO1	Forkhead Box O1
FTO	Fat Mass and Obesity-Associated Protein
GCLM	Glutamate-Cysteine Ligase Modifier Subunit
GPRC6A	G Protein-Coupled Receptor Class C Group 6 Member A
GPx	Glutathione Peroxidase
GSK3	Glycogen Synthase Kinase 3
GSTK1	Glutathione S-transferase kappa 1
HO-1	Heme Oxygenase-1
HSC70	Heat Shock Cognate 71 kDa Protein
HSP90	Heat Shock Protein 90
IDD	Intervertebral Disc Degeneration
IFN	Interferon
IGF-1	Insulin-like Growth Factor 1
IL-1α	Interleukin-1 Alpha
IL-1β	Interleukin-1 Beta
IL-6	Interleukin-6
IRF9	Interferon Regulatory Factor 9
JAK	Janus Kinase
JNK	Jun N-terminal Kinase
LC3-II	Microtubule-associated Protein 1A/1B Light Chain 3B
LPS	Lipopolysaccharide
MAPK	Mitogen-Activated Protein Kinase
MDA	Malondialdehyde
MDC1	Mediator of DNA Damage Checkpoint 1
MMP	Matrix Metalloproteinase
mTOR	Mammalian Target of Rapamycin
MyD88	Myeloid Differentiation Primary Response 88)
NAD	Nicotinamide Adenine Dinucleotide
NF-κB	Nuclear Factor Kappa-light-chain-enhancer of Activated B Cells
NLRP3	NOD-like Receptor Protein-3
NQO1	NAD(P)H Quinone Oxidoreductase 1
Nrf2	Nuclear Factor Erythroid 2-related Factor 2
p70S6K	70 kDa Ribosomal Protein S6 Kinase
PADI4	Peptidyl Arginine Deiminase 4
PCNA	Proliferating Cell Nuclear Antigen
PDE4	Phosphodiesterase 4
PGC-1α	Peroxisome Proliferator-Activated Receptor Gamma Coactivator 1-Alpha
PI3K	Phosphoinositide 3-Kinase
PINK1	PTEN-induced Kinase 1
PKA	Protein Kinase A
PTGS2	Prostaglandin Endoperoxide Synthase 2
RAGE	Receptor for Advanced Glycation Endproducts
ROS	Reactive Oxygen Species
SASP	Senescence-Associated Secretory Phenotype
SIRT1	Sirtuin 1
SOD	Superoxide Dismutase
STAT	Signal Transducers and Activators of Transcription
STING	Stimulator of Interferon Genes
TCOF1	Treacle Ribosome Biogenesis Factor 1
TERT	Telomerase Reverse Transcriptase
TGF-β1	Transforming Growth Factor beta-1
TLR2	Toll-Like Receptor 2
TNF-α	Tumor Necrosis Factor Alpha
TOM20	Translocase of Outer Mitochondrial Membrane 20
TXNIP	Thioredoxin Interacting Protein
α-MSH	α-Melanocyte Stimulating Hormone

## References

[1] Gorgoulis V., Adams P.D., Alimonti A., Bennett D.C., Bischof O., Bishop C., Campisi J., Collado M., Evangelou K., Ferbeyre G. (2019). Cellular senescence: Defining a path forward. Cell.

[2] Di Micco R., Krizhanovsky V., Baker D., d’Adda Di Fagagna F. (2021). Cellular senescence in ageing: From mechanisms to therapeutic opportunities. Nat. Rev. Mol. Cell Biol..

[3] López-Otín C., Blasco M.A., Partridge L., Serrano M., Kroemer G. (2023). Hallmarks of aging: An expanding universe. Cell.

[4] Hernandez-Segura A., Nehme J., Demaria M. (2018). Hallmarks of Cellular Senescence. Trends Cell Biol..

[5] Childs B.G., Durik M., Baker D.J., Van Deursen J.M. (2015). Cellular senescence in aging and age-related disease: From mechanisms to therapy. Nat. Med..

[6] Fulop T., Larbi A., Dupuis G., Le Page A., Frost E.H., Cohen A.A., Witkowski J.M., Franceschi C. (2018). Immunosenescence and Inflamm-Aging as Two Sides of the Same Coin: Friends or Foes?. Front. Immunol..

[7] Muñoz-Espín D., Serrano M. (2014). Cellular senescence: From physiology to pathology. Nat. Rev. Mol. Cell Biol..

[8] Saito Y., Yamamoto S., Chikenji T.S. (2024). Role of cellular senescence in inflammation and regeneration. Inflamm. Regen..

[9] Xu M., Pirtskhalava T., Farr J.N., Weigand B.M., Palmer A.K., Weivoda M.M., Inman C.L., Ogrodnik M.B., Hachfeld C.M., Fraser D.G. (2018). Senolytics improve physical function and increase lifespan in old age. Nat. Med..

[10] Baker D.J., Wijshake T., Tchkonia T., LeBrasseur N.K., Childs B.G., van de Sluis B., Kirkland J.L., van Deursen J.M. (2011). Clearance of p16^Ink4a^-positive senescent cells delays ageing-associated disorders. Nature.

[11] Baker D.J., Childs B.G., Durik M., Wijers M.E., Sieben C.J., Zhong J., Saltness R.A., Jeganathan K.B., Verzosa G.C., Pezeshki A. (2016). Naturally occurring p16^Ink4a^-positive cells shorten healthy lifespan. Nature.

[12] Baar M.P., Brandt R.M.C., Putavet D.A., Klein J.D.D., Derks K.W.J., Bourgeois B.R.M., Stryeck S., Rijksen Y., van Willigenburg H., Feijtel D.A. (2017). Targeted Apoptosis of Senescent Cells Restores Tissue Homeostasis in Response to Chemotoxicity and Aging. Cell.

[13] Cai Y., Zhou H., Zhu Y., Sun Q., Ji Y., Xue A., Wang Y., Chen W., Yu X., Wang L. (2020). Elimination of senescent cells by β-galactosidase-targeted prodrug attenuates inflammation and restores physical function in aged mice. Cell Res..

[14] Zhang L., Pitcher L.E., Prahalad V., Niedernhofer L.J., Robbins P.D. (2023). Targeting cellular senescence with senotherapeutics: Senolytics and senomorphics. FEBS J..

[15] Robbins P.D., Jurk D., Khosla S., Kirkland J.L., LeBrasseur N.K., Miller J.D., Passos J.F., Pignolo R.J., Tchkonia T., Niedernhofer L.J. (2021). Senolytic Drugs: Reducing Senescent Cell Viability to Extend Health Span. Annu. Rev. Pharmacol. Toxicol..

[16] Chang J., Wang Y., Shao L., Laberge R.-M., Demaria M., Campisi J., Janakiraman K., Sharpless N.E., Ding S., Feng W. (2016). Clearance of senescent cells by ABT263 rejuvenates aged hematopoietic stem cells in mice. Nat. Med..

[17] Yosef R., Pilpel N., Tokarsky-Amiel R., Biran A., Ovadya Y., Cohen S., Vadai E., Dassa L., Shahar E., Condiotti R. (2016). Directed elimination of senescent cells by inhibition of BCL-W and BCL-XL. Nat. Commun..

[18] Jeon O.H., Kim C., Laberge R.-M., Demaria M., Rathod S., Vasserot A.P., Chung J.W., Kim D.H., Poon Y., David N. (2017). Local clearance of senescent cells attenuates the development of post-traumatic osteoarthritis and creates a pro-regenerative environment. Nat. Med..

[19] Cherif H., Bisson D.G., Mannarino M., Rabau O., Ouellet J.A., Haglund L. (2020). Senotherapeutic drugs for human intervertebral disc degeneration and low back pain. eLife.

[20] Fuhrmann-Stroissnigg H., Ling Y.Y., Zhao J., McGowan S.J., Zhu Y., Brooks R.W., Grassi D., Gregg S.Q., Stripay J.L., Dorronsoro A. (2017). Identification of HSP90 inhibitors as a novel class of senolytics. Nat. Commun..

[21] Moiseeva O., Deschênes-Simard X., St-Germain E., Igelmann S., Huot G., Cadar A.E., Bourdeau V., Pollak M.N., Ferbeyre G. (2013). Metformin inhibits the senescence-associated secretory phenotype by interfering with IKK/NF-κB activation. Aging Cell.

[22] Selvarani R., Mohammed S., Richardson A. (2021). Effect of rapamycin on aging and age-related diseases—Past and future. GeroScience.

[23] Hubbard B.P., Sinclair D.A. (2014). Small molecule SIRT1 activators for the treatment of aging and age-related diseases. Trends Pharmacol. Sci..

[24] Gandhi L., Camidge D.R., Ribeiro De Oliveira M., Bonomi P., Gandara D., Khaira D., Hann C.L., McKeegan E.M., Litvinovich E., Hemken P.M. (2011). Phase I Study of Navitoclax (ABT-263), a Novel Bcl-2 Family Inhibitor, in Patients with Small-Cell Lung Cancer and Other Solid Tumors. J. Clin. Oncol..

[25] Nambiar A., Kellogg D., Justice J., Goros M., Gelfond J., Pascual R., Hashmi S., Masternak M., Prata L., LeBrasseur N. (2023). Senolytics dasatinib and quercetin in idiopathic pulmonary fibrosis: Results of a phase I, single-blind, single-center, randomized, placebo-controlled pilot trial on feasibility and tolerability. eBioMedicine.

[26] Yousefzadeh M.J., Zhu Y., McGowan S.J., Angelini L., Fuhrmann-Stroissnigg H., Xu M., Ling Y.Y., Melos K.I., Pirtskhalava T., Inman C.L. (2018). Fisetin is a senotherapeutic that extends health and lifespan. eBioMedicine.

[27] Zhu Y., Tchkonia T., Pirtskhalava T., Gower A.C., Ding H., Giorgadze N., Palmer A.K., Ikeno Y., Hubbard G.B., Lenburg M. (2015). The Achilles’ heel of senescent cells: From transcriptome to senolytic drugs. Aging Cell.

[28] Li W., He Y., Zhang R., Zheng G., Zhou D. (2019). The curcumin analog EF24 is a novel senolytic agent. Aging.

[29] Lim H., Park H., Kim H.P. (2015). Effects of flavonoids on senescence-associated secretory phenotype formation from bleomycin-induced senescence in BJ fibroblasts. Biochem. Pharmacol..

[30] Zumerle S., Sarill M., Saponaro M., Colucci M., Contu L., Lazzarini E., Sartori R., Pezzini C., Rinaldi A., Scanu A. (2024). Targeting senescence induced by age or chemotherapy with a polyphenol-rich natural extract improves longevity and healthspan in mice. Nat. Aging.

[31] Centonze M., Aloisio Caruso E., De Nunzio V., Cofano M., Saponara I., Pinto G., Notarnicola M. (2025). The Antiaging Potential of Dietary Plant-Based Polyphenols: A Review on Their Role in Cellular Senescence Modulation. Nutrients.

[32] Proshkina E., Koval L., Platonova E., Golubev D., Ulyasheva N., Babak T., Shaposhnikov M., Moskalev A. (2024). Polyphenols as Potential Geroprotectors. Antioxid. Redox Signal..

[33] Della Vedova L., Baron G., Morazzoni P., Aldini G., Gado F. (2025). The Potential of Polyphenols in Modulating the Cellular Senescence Process: Implications and Mechanism of Action. Pharmaceuticals.

[34] Proshkina E., Plyusnin S., Babak T., Lashmanova E., Maganova F., Koval L., Platonova E., Shaposhnikov M., Moskalev A. (2020). Terpenoids as Potential Geroprotectors. Antioxidants.

[35] Mazzone V., Alessio N., Aprile D., Galano G., De Rosa R., Schiraldi C., Di Bernardo G., Galderisi U. (2025). Terpenes: Natural compounds found in plants as potential senotherapeutics targeting senescent mesenchymal stromal cells and promoting apoptosis. Stem Cell Res. Ther..

[36] Fakhri S., Moradi S.Z., Ash-Rafzadeh A., Bishayee A. (2022). Targeting cellular senescence in cancer by plant secondary metabolites: A systematic review. Pharmacol. Res..

[37] Câmara J.S., Perestrelo R., Ferreira R., Berenguer C.V., Pereira J.A.M., Castilho P.C. (2024). Plant-Derived Terpenoids: A Plethora of Bioactive Compounds with Several Health Functions and Industrial Applications—A Comprehensive Overview. Molecules.

[38] Zeng T., Liu Z., Liu H., He W., Tang X., Xie L., Wu R. (2019). Exploring Chemical and Biological Space of Terpenoids. J. Chem. Inf. Model..

[39] Kumar K.J.S., Vani M.G., Wang S.-Y. (2022). Limonene protects human skin keratinocytes against UVB-induced photodamage and photoaging by activating the Nrf2-dependent antioxidant defense system. Environ. Toxicol..

[40] AlBairmani R.J.H., Shihab E.M., Ridha-Salman H., Kadhim H.M., Mohammed R.M., Shahooth S.S., Abdalah M.E., Hameed Z.E., Hajwal S.K., Albasri O.W. (2025). D-limonene attenuates D-galactose-induced skin aging mouse model. J. Mol. Histol..

[41] Civiletto G., Brunetti D., Lizzo G., Muller K., Jacot G.E., Daskalaki I., Sizzano F., Huh M., Di Meo I., Colombo M.N. (2025). Herbal terpenoids activate autophagy and mitophagy through modulation of bioenergetics and protect from metabolic stress, sarcopenia and epigenetic aging. Nat. Aging.

[42] Deng J., Luo C., Xie C., Duan H. (2025). Thymol Mitigates Oxidative Stress-Induced Ovarian Aging and Restores Steroidogenesis via the JAK1–STAT3 Pathway. Curr. Issues Mol. Biol..

[43] Wan X., Li C., Tan Y.H., Zuo S.Q., Deng F.M., Sun J., Liu Y.L. (2024). Dihydroartemisinin eliminates senescent cells by promoting autophagy-dependent ferroptosis via AMPK/mTOR signaling pathway. Cell Biol. Int..

[44] Li D., Qi J., Wang J., Pan Y., Li J., Xia X., Dou H., Hou Y. (2019). Protective effect of dihydroartemisinin in inhibiting senescence of myeloid-derived suppressor cells from lupus mice via Nrf2/HO-1 pathway. Free Radic. Biol. Med..

[45] Liao Z., Su D., Liu H., Xu C., Wu J., Chen Y., Guo W., Zhang S., Li Z., Ke X. (2022). Dihydroartemisinin Attenuated Intervertebral Disc Degeneration via Inhibiting PI3K/AKT and NF-κB Signaling Pathways. Oxidative Med. Cell. Longev..

[46] Liu H., Huang Z., Jiang H., Su K., Si Z., Wu W., Wang H., Li D., Tan N., Zhang Z. (2023). Dihydroartemisinin attenuates ischemia/reperfusion-induced renal tubular senescence by activating autophagy. Chin. J. Nat. Med..

[47] Zeng Q., Feng Y., Huang H., Zou K., Chen W., Li X., Huang Y., Wang W., Yuan W., Wang P. (2025). Dihydroartemisinin ameliorates hemarthrosis-induced cartilage degeneration by suppressing chondrocyte senescence via activation of Keap1-Nrf2 signaling pathway. J. Orthop. Transl..

[48] Sun S., Jiang P., Su W., Xiang Y., Li J., Zeng L., Yang S. (2016). Wild chrysanthemum extract prevents UVB radiation-induced acute cell death and photoaging. Cytotechnology.

[49] Zhang H.-J., Wang B.-H., Wang X., Huang C.-P., Xu S.-M., Wang J.-L., Huang T.-E., Xiao W.-L., Tian X.-L., Lan X.-Q. (2024). Handelin alleviates cachexia- and aging-induced skeletal muscle atrophy by improving protein homeostasis and inhibiting inflammation. J. Cachexia Sarcopenia Muscle.

[50] Pant A., Saikia S.K., Shukla V., Asthana J., Akhoon B.A., Pandey R. (2014). Beta-caryophyllene modulates expression of stress response genes and mediates longevity in *Caenorhabditis elegans*. Exp. Gerontol..

[51] Matacchione G., Gurău F., Silvestrini A., Tiboni M., Mancini L., Valli D., Rippo M.R., Recchioni R., Marcheselli F., Carnevali O. (2021). Anti-SASP and anti-inflammatory activity of resveratrol, curcumin and β-caryophyllene association on human endothelial and monocytic cells. Biogerontology.

[52] Lee C.-W., Wang B.Y.-H., Wong S.H., Chen Y.-F., Cao Q., Hsiao A.W.-T., Fung S.-H., Chen Y.-F., Wu H.-H., Cheng P.-Y. (2025). Ginkgolide B increases healthspan and lifespan of female mice. Nat. Aging.

[53] Wang B.Y.-H., Chen Y.-F., Hsiao A.W.-T., Chen W.-J., Lee C.-W., Lee O.K.-S. (2023). Ginkgolide B facilitates muscle regeneration via rejuvenating osteocalcin-mediated bone-to-muscle modulation in aged mice. J. Cachexia Sarcopenia Muscle.

[54] Zhang Y., Zhang Q., Chu Z., Chen L., Chen J., Yang Y., Tang H., Cheng G., Ma A., Zhang Y. (2022). Oridonin acts as a novel senolytic by targeting glutathione S-transferases to activate the ROS-p38 signaling axis in senescent cells. Chem. Commun..

[55] Yasuda S., Horinaka M., Iizumi Y., Goi W., Sukeno M., Sakai T. (2022). Oridonin inhibits SASP by blocking p38 and NF-κB pathways in senescent cells. Biochem. Biophys. Res. Commun..

[56] An Y., Zhu J., Wang X., Sun X., Luo C., Zhang Y., Ye Y., Li X., Abulizi A., Huang Z. (2022). Oridonin Delays Aging Through the AKT Signaling Pathway. Front. Pharmacol..

[57] Cao D., Zhang M., Jiang C., Xue L., Sun C. (2012). Protection of Tanshinone IIA to human peritoneal mesothelial cells (HPMC) through delaying cellular senescence induced by high glucose. Ren. Fail..

[58] Xiao X., Xu M., Fang Y. (2019). Tanshinone IIA attenuates hydrogen peroxide-induced senescence of human umbilical vein endothelial cells through activating SIRT1/eNOS pathway. Chin. J. Cell. Mol. Immunol..

[59] Wei W., Heng Y.-Y., Wu F.-F., Dong H.-Y., Zhang P.-F., Li J.-X., Liu C.-Y., Yang B.-J., Fu J.-N., Liang X.-Y. (2024). Sodium Tanshinone IIA Sulfonate alleviates vascular senescence in diabetic mice by modulating the A20-NFκB-NLRP3 inflammasome-catalase pathway. Sci. Rep..

[60] Heng Y.-Y., Shang H.-J., Zhang X.-Z., Wei W. (2023). Sodium tanshinone IIA sulfonate ameliorates neointima by protecting endothelial progenitor cells in diabetic mice. BMC Cardiovasc. Disord..

[61] Yang M., Wu S., Dai Q., Qin W., Zhang Y., Lei Y., Song H., Zheng T., Guan M., Huang G. (2024). Andrographolide prevents renal fibrosis via decelerating lipotoxicity-mediated premature senescence of tubular epithelial cells. Biochem. Pharmacol..

[62] Chen M., Zhou S., Sun K., Hu Y., Wen H., Ren G. (2023). Andrographolide protects BMSCs from dexamethasone-induced cellular dysfunction and promotes bone formation via the PI3K/AKT pathway. J. Funct. Foods.

[63] Wei H., Liu Y., Huang C., Wang C., Jiang H., Wang L., Wang Y., Wang Z. (2025). Ginsenoside Rg1 targets TLR2 to inhibit the NF-κB signaling pathway and ameliorate hematopoietic support of mesenchymal stromal cells. J. Ethnopharmacol..

[64] Wang Z., Jiang R., Wang L., Chen X., Xiang Y., Chen L., Xiao M., Ling L., Wang Y. (2020). Ginsenoside Rg1 Improves Differentiation by Inhibiting senescence of Human Bone Marrow Mesenchymal Stem Cell via GSK-3β and β-Catenin. Stem Cells Int..

[65] Wang Z., Wang L., Jiang R., Li C., Chen X., Xiao H., Hou J., Hu L., Huang C., Wang Y. (2021). Ginsenoside Rg1 prevents bone marrow mesenchymal stem cell senescence via NRF2 and PI3K/Akt signaling. Free Radic. Biol. Med..

[66] Fang Y., Mou C., Yu X., Zhang J., Zhu W., Zhou C., Jiang B., Chen Y. (2026). Ginsenoside Rg1 delays the senescence of adipose-derived stem cells: Network pharmacology and experimental validation. Hereditas.

[67] Chen L., Yao H., Chen X., Wang Z., Xiang Y., Xia J., Liu Y., Wang Y. (2018). Ginsenoside Rg1 Decreases Oxidative Stress and Down-Regulates Akt/mTOR Signalling to Attenuate Cognitive Impairment in Mice and Senescence of Neural Stem Cells Induced by d-Galactose. Neurochem. Res..

[68] Hu L., Ran J., Wang L., Wu M., Wang Z., Xiao H., Du K., Wang Y. (2023). Ginsenoside Rg1 attenuates D-galactose-induced neural stem cell senescence via the Sirt1-Nrf2-BDNF pathway. Eur. J. Neurosci..

[69] Huang C., Xue X., Gong N., Jiang J. (2022). Ginsenoside Rg1 suppresses paraquat-induced epithelial cell senescence by enhancing autophagy in an ATG12-dependent manner. Environ. Toxicol..

[70] Ding R., Xie H., Zhang Y., Qin L., Peng G., Yi J., Tan J., Cao X., Zheng R., Dai A. (2025). Ginsenoside Rg1 Ameliorates Pulmonary Hypertension by Inhibiting cGAS/STING Mediated Cell Senescence. Drug Des. Dev. Ther..

[71] Shen X., Dong X., Han Y., Li Y., Ding S., Zhang H., Sun Z., Yin Y., Li W., Li W. (2020). Ginsenoside Rg1 ameliorates glomerular fibrosis during kidney aging by inhibiting NOX4 and NLRP3 inflammasome activation in SAMP8 mice. Int. Immunopharmacol..

[72] Guo T., Xie T., Chen X., Zhong J., Bai Z., Liang H., Zhao X., Fang M., Xiao Y., Zhang J. (2025). Inhibition of caspase-1 by ginsenoside Rg1 ameliorates d-gal-induced renal aging and injury through suppression of oxidative stress and inflammation. Ren. Fail..

[73] Qi R., Jiang R., Xiao H., Wang Z., He S., Wang L., Wang Y. (2020). Ginsenoside Rg1 protects against d-galactose induced fatty liver disease in a mouse model via FOXO1 transcriptional factor. Life Sci..

[74] Tang K., Huang C., Huang Z., Lin Z., Wang Z., Tan N. (2026). Ginsenoside Rg1 ameliorates the senescence and neurite injury of retinal ganglion cells in DR via targeting VDR and promoting mitochondrial biogenesis. J. Ginseng Res..

[75] Xia M.-L., Xie X.-H., Ding J.-H., Du R.-H., Hu G. (2020). Astragaloside IV inhibits astrocyte senescence: Implication in Parkinson’s disease. J. Neuroinflamm..

[76] Li H., Xu J., Zhang Y., Hong L., He Z., Zeng Z., Zhang L. (2022). Astragaloside IV alleviates senescence of vascular smooth muscle cells through activating Parkin-mediated mitophagy. Hum. Cell.

[77] Ding Y., Jiang C., Chen L., Liu X., Shao B. (2025). Astragaloside IV confers neuroprotection against radiation-induced neuronal senescence via the ERK pathway. Exp. Neurol..

[78] Yan X., Zeng R., Cao Y. (2024). Astragaloside IV confronts amyloid-beta-induced astrocyte senescence via hsp90aa1. J. Appl. Biomed..

[79] Li M., Niu Y., Tian L., Zhang T., Zhou S., Wang L., Sun J., Wumiti T., Chen Z., Zhou Q. (2024). Astragaloside IV alleviates macrophage senescence and d-galactose-induced bone loss in mice through STING/NF-κB pathway. Int. Immunopharmacol..

[80] Wang S.-W., Li P., Liu S.-Y., Huang D.-L., Zhang S.-J., Zeng X.-X., Lan T., Mao K.-L., Gao Y., Cheng Y.-F. (2025). Astragaloside IV inhibits retinal pigment epithelial cell senescence and reduces IL-1β mRNA stability by targeting FTO-mediated m6A methylation. Phytomedicine.

[81] Xu W., Chen H., Gong H., Zhao T., Yang Y., Wang F., Huang N., Yang M., Gao N., Li H. (2026). Celastrol Targets Hsc70-Bim Interaction as a Novel Senolytic to Extend Lifespan and Mitigate Organ Fibrosis. Phytother. Res..

[82] Zhang S., Zhu N., Shi Y.-N., Zeng Q., Zhang C.-J., Li H.-F., Qin L. (2024). Celastrol mediates CAV1 to attenuate pro-tumorigenic effects of senescent cells. Phytomedicine.

[83] Zhang Y., Sun Z., Meng W., Lei S., Sun J., Tang Y., Mu X. (2025). Celastrol attenuates diabetic kidney disease progression by repressing senescence of renal tubular epithelial cells. Front. Aging.

[84] Lissa D., Joruiz S.M., Dranchak P.K., Ungerleider K., Yamada L., Horikawa I., Oliphant E., Manubag R., Von Muhlinen N., Beck J. (2025). A Quantitative High-Throughput Screen Identifies Compounds that Upregulate the p53 Isoform Δ133p53α and Inhibit Cellular Senescence. ACS Pharmacol. Transl. Sci..

[85] Xu X.-J., Zhao W.-B., Feng S.-B., Sun C., Chen Q., Ni B., Hu H.-Y. (2017). Celastrol alleviates angiotensin II-mediated vascular smooth muscle cell senescence via induction of autophagy. Mol. Med. Rep..

[86] Chen L., Wu B., Mo L., Chen H., Yin X., Zhao Y., Cui Z., Cui F., Chen L., Deng Q. (2025). High-content screening identifies ganoderic acid A as a senotherapeutic to prevent cellular senescence and extend healthspan in preclinical models. Nat. Commun..

[87] Shen S., Wang X., Lv H., Shi Y., Xiao L. (2021). PADI4 mediates autophagy and participates in the role of ganoderic acid A monomers in delaying the senescence of Alzheimer’s cells through the Akt/mTOR pathway. Biosci. Biotechnol. Biochem..

[88] Yuan H., Xu Y., Luo Y., Zhang J.R., Zhu X.X., Xiao J.H. (2022). Ganoderic acid D prevents oxidative stress-induced senescence by targeting 14-3-3ε to activate CaM/CaMKII/NRF2 signaling pathway in mesenchymal stem cells. Aging Cell.

[89] Xu Y., Yuan H., Luo Y., Zhao Y.-J., Xiao J.-H. (2020). Ganoderic Acid D Protects Human Amniotic Mesenchymal Stem Cells against Oxidative Stress-Induced Senescence through the PERK/NRF2 Signaling Pathway. Oxidative Med. Cell. Longev..

[90] Zhang Y., Gao D., Yuan Y., Zheng R., Sun M., Jia S., Liu J. (2023). Cycloastragenol: A Novel Senolytic Agent That Induces Senescent Cell Apoptosis and Restores Physical Function in TBI-Aged Mice. Int. J. Mol. Sci..

[91] Weng W., Lin B., Zheng J., Sun Y., Li Z., Chen X., Wang Y., Pan X. (2025). Novel application of cycloastragenol target microglia for the treatment of Alzheimer’s disease: Evidence from single-cell analysis, network pharmacology and experimental assessment. Phytomedicine.

[92] Hong H., Xiao J., Guo Q., Du J., Jiang Z., Lu S., Zhang H., Zhang X., Wang X. (2021). Cycloastragenol and Astragaloside IV activate telomerase and protect nucleus pulposus cells against high glucose-induced senescence and apoptosis. Exp. Ther. Med..

[93] Bai S.-R., Zhao B.-X., Zhao Q., Ge Y.-C., Li M., Zhao C.-G., Wu X.-J., Wang X.-B. (2024). Oleanolic acid improves 5-fluorouracil-induced intestinal damage and inflammation by alleviating intestinal senescence. Sci. Rep..

[94] Xu Y., Wei J., Wang W., Mao Z., Wang D., Zhang T., Zhang P. (2025). Oleanolic Acid Slows Down Aging Through IGF-1 Affecting the PI3K/AKT/mTOR Signaling Pathway. Molecules.

[95] Gao H., Liu T., Liu J., Yang L., Liu L., Cui Z., Du X., Gu Y., Huang P. (2025). Oleanolic Acid@SPIONs Alleviates Lipid-Oxidative Stress Injury of Zebrafish Blood Vessels via Regulating the Expression of JNK and MAPK Signaling Pathways in Vascular Endothelial Cells. Drug Des. Dev. Ther..

[96] Odama M., Maegawa E., Suzuki K., Fujii Y., Maeda R., Murakami S., Ito T. (2023). Effects of Betulinic Acid on the Proliferation, Cellular Senescence, and Type 1 Interferon-Related Signaling Pathways in Human Dermal Fibroblasts. J. Agric. Food Chem..

[97] Chen H., Li R., Ma X., Qiu Z., Qiu Y. (2024). Network pharmacology and experimental validation to explore the anti-aging mechanism of action of betulinic acid. Netw. Model. Anal. Health Inform. Bioinform..

[98] Anandakumar P., Kamaraj S., Vanitha M.K. (2021). D-limonene: A multifunctional compound with potent therapeutic effects. J. Food Biochem..

[99] Gad H.A., El Hassab M.A., Elhady S.S., Fahmy N.M. (2023). Insights on *Citrus clementina* essential oil as a potential antiaging candidate with a comparative chemometric study on different cultivars. Ind. Crops Prod..

[100] Shukla P., Pant A., Pandey R. (2019). Limonene attenuates oxidative stress and extends longevity in *Caenorhabditis elegans*. Curr. Sci..

[101] Silva B.I.M., Nascimento E.A., Silva C.J., Silva T.G., Aguiar J.S. (2021). Anticancer activity of monoterpenes: A systematic review. Mol. Biol. Rep..

[102] Jiang C., Li S., Li Y., Bai Y. (2018). Anticancer Effects of Dihydroartemisinin on Human Esophageal Cancer Cells In Vivo. Anal. Cell. Pathol..

[103] Yang D.X., Qiu J., Zhou H.H., Yu Y., Zhou D.L., Xu Y., Zhu M.Z., Ge X.P., Li J.M., Lv C.J. (2018). Dihydroartemisinin alleviates oxidative stress in bleomycin-induced pulmonary fibrosis. Life Sci..

[104] Ooko E., Saeed M.E.M., Kadioglu O., Sarvi S., Colak M., Elmasaoudi K., Janah R., Greten H.J., Efferth T. (2015). Artemisinin derivatives induce iron-dependent cell death (ferroptosis) in tumor cells. Phytomedicine.

[105] Shen J., Bin W., Lin X., Lai Y., Lin X., Guan T., Liu H. (2025). Klotho Protein: A Multifaceted Guardian of Healthy Aging and Its Therapeutic Potential. Int. J. Nanomed..

[106] Zhang H., Qin J., Lan X., Zeng W., Zhou J., Huang T.-E., Xiao W.-L., Wang Q.-Q., Sun S., Su W. (2022). Handelin extends lifespan and healthspan of *Caenorhabditis elegans* by reducing ROS generation and improving motor function. Biogerontology.

[107] Wang L.C., Liao L.X., Lv H.N., Liu D., Dong W., Zhu J., Chen J.F., Shi M.L., Fu G., Song X.M. (2017). Highly Selective Activation of Heat Shock Protein 70 by Allosteric Regulation Provides an Insight into Efficient Neuroinflammation Inhibition. eBioMedicine.

[108] Gertsch J., Leonti M., Raduner S., Racz I., Chen J.-Z., Xie X.-Q., Altmann K.-H., Karsak M., Zimmer A. (2008). Beta-caryophyllene is a dietary cannabinoid. Proc. Natl. Acad. Sci. USA.

[109] Calleja M.A., Vieites J.M., Montero-Meterdez T., Torres M.I., Faus M.J., Gil A., Suárez A. (2013). The antioxidant effect of β-caryophyllene protects rat liver from carbon tetrachloride-induced fibrosis by inhibiting hepatic stellate cell activation. Br. J. Nutr..

[110] Legault J., Pichette A. (2007). Potentiating effect of β-caryophyllene on anticancer activity of α-humulene, isocaryophyllene and paclitaxel. J. Pharm. Pharmacol..

[111] Xia S.-H., Fang D.-C. (2007). Pharmacological action and mechanisms of ginkgolide B. Chin. Med. J..

[112] Zhao G., Zhang T., Ma X., Jiang K., Wu H., Qiu C., Guo M., Deng G. (2017). Oridonin attenuates the release of pro-inflammatory cytokines in lipopolysaccharide-induced RAW264.7 cells and acute lung injury. Oncotarget.

[113] Bae S., Lee E.J., Lee J.H., Park I.C., Lee S.J., Hahn H.J., Ahn K.J., An S., An I.S., Cha H.J. (2014). Oridonin protects HaCaT keratinocytes against hydrogen peroxide-induced oxidative stress by altering microRNA expression. Int. J. Mol. Med..

[114] Li C.Y., Wang E.Q., Cheng Y., Bao J.K. (2011). Oridonin: An active diterpenoid targeting cell cycle arrest, apoptotic and autophagic pathways for cancer therapeutics. Int. J. Biochem. Cell Biol..

[115] Qin Y., Zheng B., Yang G.-S., Zhou J., Yang H.-J., Nie Z.-Y., Wang T.-R., Zhang X.-H., Zhao H.-Y., Shi J.-H. (2020). Tanshinone II A inhibits VSMC inflammation and proliferation in vivo and in vitro by downregulating miR-712-5p expression. Eur. J. Pharmacol..

[116] Tan W.S.D., Liao W., Zhou S., Wong W.S.F. (2017). Is there a future for andrographolide to be an anti-inflammatory drug? Deciphering its major mechanisms of action. Biochem. Pharmacol..

[117] Ji X., Li C., Ou Y., Li N., Yuan K., Yang G., Chen X., Yang Z., Liu B., Cheung W.W. (2016). Andrographolide ameliorates diabetic nephropathy by attenuating hyperglycemia-mediated renal oxidative stress and inflammation via Akt/NF-κB pathway. Mol. Cell. Endocrinol..

[118] Lee C.H., Kim J.-H. (2014). A review on the medicinal potentials of ginseng and ginsenosides on cardiovascular diseases. J. Ginseng Res..

[119] Wu J.-J., Yang Y., Wan Y., Xia J., Xu J.-F., Zhang L., Liu D., Chen L., Tang F., Ao H. (2022). New insights into the role and mechanisms of ginsenoside Rg1 in the management of Alzheimer’s disease. Biomed. Pharmacother..

[120] Zarneshan S.N., Fakhri S., Khan H. (2022). Targeting Akt/CREB/BDNF signaling pathway by ginsenosides in neurodegenerative diseases: A mechanistic approach. Pharmacol. Res..

[121] Xie W., Zhou P., Sun Y., Meng X., Dai Z., Sun G., Sun X. (2018). Protective Effects and Target Network Analysis of Ginsenoside Rg1 in Cerebral Ischemia and Reperfusion Injury: A Comprehensive Overview of Experimental Studies. Cells.

[122] Bai L., Gao J., Wei F., Zhao J., Wang D., Wei J. (2018). Therapeutic Potential of Ginsenosides as an Adjuvant Treatment for Diabetes. Front. Pharmacol..

[123] Gao Y., Chu S., Zhang Z., Chen N. (2017). Hepataprotective effects of ginsenoside Rg1—A review. J. Ethnopharmacol..

[124] Gao Y., Li J., Wang J., Li X., Li J., Chu S., Li L., Chen N., Zhang L. (2020). Ginsenoside Rg1 prevent and treat inflammatory diseases: A review. Int. Immunopharmacol..

[125] Kim J.H., Yi Y.-S., Kim M.-Y., Cho J.Y. (2017). Role of ginsenosides, the main active components of *Panax ginseng*, in inflammatory responses and diseases. J. Ginseng Res..

[126] Cheng Y., Shen L.-H., Zhang J.-T. (2005). Anti-amnestic and anti-aging effects of ginsenoside Rg1 and Rb1 and its mechanism of action. Acta Pharmacol. Sin..

[127] Shang D., Li Z., Tan X., Liu H., Tu Z. (2023). Inhibitory effects and molecular mechanisms of ginsenoside Rg1 on the senescence of hematopoietic stem cells. Fundam. Clin. Pharmacol..

[128] Chen C., Mu X.-Y., Zhou Y., Shun K., Geng S., Liu J., Wang J.-W., Chen J., Li T.-Y., Wang Y.-P. (2014). Ginsenoside Rg1 enhances the resistance of hematopoietic stem/progenitor cells to radiation-induced aging in mice. Acta Pharmacol. Sin..

[129] Flohr Svendsen A., Yang D., Kim K., Lazare S., Skinder N., Zwart E., Mura-Meszaros A., Ausema A., Von Eyss B., De Haan G. (2021). A comprehensive transcriptome signature of murine hematopoietic stem cell aging. Blood.

[130] Zhou X., Hong Y., Zhang H., Li X. (2020). Mesenchymal Stem Cell Senescence and Rejuvenation: Current Status and Challenges. Front. Cell Dev. Biol..

[131] Gu Z., Tan W., Feng G., Meng Y., Shen B., Liu H., Cheng C. (2014). Wnt/β-catenin signaling mediates the senescence of bone marrow-mesenchymal stem cells from systemic lupus erythematosus patients through the p53/p21 pathway. Mol. Cell. Biochem..

[132] Li L., Gan H., Jin H., Fang Y., Yang Y., Zhang J., Hu X., Chu L. (2021). Astragaloside IV promotes microglia/macrophages M2 polarization and enhances neurogenesis and angiogenesis through PPARγ pathway after cerebral ischemia/reperfusion injury in rats. Int. Immunopharmacol..

[133] Yao M., Zhang L., Wang L. (2023). Astragaloside IV: A promising natural neuroprotective agent for neurological disorders. Biomed. Pharmacother..

[134] Zhang Y., Zhang Y., Jin X.-F., Zhou X.-H., Dong X.-H., Yu W.-T., Gao W.-J. (2019). The Role of Astragaloside IV against Cerebral Ischemia/Reperfusion Injury: Suppression of Apoptosis via Promotion of P62-LC3-Autophagy. Molecules.

[135] Zhang X., Qu H., Yang T., Liu Q., Zhou H. (2022). Astragaloside IV attenuate MI-induced myocardial fibrosis and cardiac remodeling by inhibiting ROS/caspase-1/GSDMD signaling pathway. Cell Cycle.

[136] Luo L.-F., Guan P., Qin L.-Y., Wang J.-X., Wang N., Ji E.-S. (2021). Astragaloside IV inhibits adriamycin-induced cardiac ferroptosis by enhancing Nrf2 signaling. Mol. Cell. Biochem..

[137] Chen M., Fu B., Zhou H., Wu Q. (2025). Therapeutic potential and mechanistic insights of astragaloside IV in the treatment of arrhythmia: A comprehensive review. Front. Pharmacol..

[138] Liu H., Wei W., Sun W.-Y., Li X. (2009). Protective effects of astragaloside IV on porcine-serum-induced hepatic fibrosis in rats and in vitro effects on hepatic stellate cells. J. Ethnopharmacol..

[139] Liu X., Shang S., Chu W., Ma L., Jiang C., Ding Y., Wang J., Zhang S., Shao B. (2020). Astragaloside IV ameliorates radiation-induced senescence via antioxidative mechanism. J. Pharm. Pharmacol..

[140] Yuan S., Zuo B., Zhou S.-C., Wang M., Tan K.-Y., Chen Z.-W., Cao W.-F. (2023). Integrating Network Pharmacology and Experimental Validation to Explore the Pharmacological Mechanism of Astragaloside IV in Treating Bleomycin-Induced Pulmonary Fibrosis. Drug Des. Dev. Ther..

[141] Shi L., Deng J., He J., Zhu F., Jin Y., Zhang X., Ren Y., Du X. (2024). Integrative transcriptomics and proteomics analysis reveal the protection of Astragaloside IV against myocardial fibrosis by regulating senescence. Eur. J. Pharmacol..

[142] Schafer M.J., White T.A., Iijima K., Haak A.J., Ligresti G., Atkinson E.J., Oberg A.L., Birch J., Salmonowicz H., Zhu Y. (2017). Cellular senescence mediates fibrotic pulmonary disease. Nat. Commun..

[143] Guo Q., Chen X., Chen J., Zheng G., Xie C., Wu H., Miao Z., Lin Y., Wang X., Gao W. (2021). STING promotes senescence, apoptosis, and extracellular matrix degradation in osteoarthritis via the NF-κB signaling pathway. Cell Death Discov..

[144] Wang C., Dai S., Zhao X., Zhang Y., Gong L., Fu K., Ma C., Peng C., Li Y. (2023). Celastrol as an emerging anticancer agent: Current status, challenges and therapeutic strategies. Biomed. Pharmacother..

[145] Meng Z., Liu Q., Chen H., She C., Huang Y. (2025). Therapeutic potential of celastrol in bacterial infections: Current research advancements and future perspectives. Pharmacol. Res..

[146] Volonte D., Galbiati F. (2020). Caveolin-1, a master regulator of cellular senescence. Cancer Metastasis Rev..

[147] Von Muhlinen N., Horikawa I., Alam F., Isogaya K., Lissa D., Vojtesek B., Lane D.P., Harris C.C. (2018). p53 isoforms regulate premature aging in human cells. Oncogene.

[148] Horikawa I., Park K.-Y., Isogaya K., Hiyoshi Y., Li H., Anami K., Robles A.I., Mondal A.M., Fujita K., Serrano M. (2017). Δ133p53 represses p53-inducible senescence genes and enhances the generation of human induced pluripotent stem cells. Cell Death Differ..

[149] Wang J., Cao B., Zhao H., Feng J. (2017). Emerging Roles of *Ganoderma lucidum* in Anti-Aging. Aging Dis..

[150] Liang C., Tian D., Liu Y., Li H., Zhu J., Li M., Xin M., Xia J. (2019). Review of the molecular mechanisms of *Ganoderma lucidum* triterpenoids: Ganoderic acids A, C2, D, F, DM, X and Y. Eur. J. Med. Chem..

[151] Lessard F., Igelmann S., Trahan C., Huot G., Saint-Germain E., Mignacca L., Del Toro N., Lopes-Paciencia S., Le Calve B., Montero M. (2018). Senescence-associated ribosome biogenesis defects contributes to cell cycle arrest through the Rb pathway. Nat. Cell. Biol..

[152] Sloan K.E., Bohnsack M.T., Watkins N.J. (2013). The 5S RNP couples p53 homeostasis to ribosome biogenesis and nucleolar stress. Cell Rep..

[153] Nishimura K., Kumazawa T., Kuroda T., Katagiri N., Tsuchiya M., Goto N., Furumai R., Murayama A., Yanagisawa J., Kimura K. (2015). Perturbation of ribosome biogenesis drives cells into senescence through 5S RNP-mediated p53 activation. Cell Rep..

[154] Abdelmoaty A.A.A., Chen J., Zhang K., Wu C., Li Y., Li P., Xu J. (2024). Senolytic effect of triterpenoid complex from *Ganoderma lucidum* on adriamycin-induced senescent human hepatocellular carcinoma cells model in vitro and in vivo. Front. Pharmacol..

[155] Kühnel H., Seiler M., Feldhofer B., Ebrahimian A., Maurer M. (2025). *Ganoderma lucidum* Extract Modulates Gene Expression Profiles Associated with Antioxidant Defense, Cytoprotection, and Senescence in Human Dermal Fibroblasts: Investigation of Quantitative Gene Expression by qPCR. Curr. Issues Mol. Biol..

[156] Yu Y., Zhou L., Yang Y., Liu Y. (2018). Cycloastragenol: An exciting novel candidate for age-associated diseases. Exp. Ther. Med..

[157] Yilmaz S., Bedir E., Ballar Kirmizibayrak P. (2022). The role of cycloastragenol at the intersection of NRF2/ARE, telomerase, and proteasome activity. Free Radic. Biol. Med..

[158] Liu J., Gao D., Dan J., Liu D., Peng L., Zhou R., Luo Y. (2019). The protective effect of cycloastragenol on aging mouse circadian rhythmic disorder induced by D-galactose. J. Cell. Biochem..

[159] Zhao C., Yang X., Yao M., Song X., Dai J., He P. (2026). Cycloastragenol in inflammation-related diseases: Mechanisms, pharmacokinetics, and translational prospects. Front. Pharmacol..

[160] Ayeleso T., Matumba M., Mukwevho E. (2017). Oleanolic Acid and Its Derivatives: Biological Activities and Therapeutic Potential in Chronic Diseases. Molecules.

[161] Wasim M., Bergonzi M.C. (2024). Unlocking the Potential of Oleanolic Acid: Integrating Pharmacological Insights and Advancements in Delivery Systems. Pharmaceutics.

[162] Ou Z., Zhao J., Zhu L., Huang L., Ma Y., Ma C., Luo C., Zhu Z., Yuan Z., Wu J. (2019). Anti-inflammatory effect and potential mechanism of betulinic acid on λ-carrageenan-induced paw edema in mice. Biomed. Pharmacother..

[163] Wang D., Chen T., Liu F. (2018). Betulinic acid alleviates myocardial hypoxia/reoxygenation injury via inducing Nrf2/HO-1 and inhibiting p38 and JNK pathways. Eur. J. Pharmacol..

[164] Fulda S. (2009). Betulinic acid: A natural product with anticancer activity. Mol. Nutr. Food Res..

[165] Sudharshan S.J., Krishna Narayanan A., Princilly J., Dyavaiah M., Nagegowda D.A. (2022). Betulinic acid mitigates oxidative stress-mediated apoptosis and enhances longevity in the yeast *Saccharomyces cerevisiae* model. Free Radic. Res..

[166] Chen H., Li R., Zhao F., Luan L., Han T., Li Z. (2022). Betulinic acid increases lifespan and stress resistance via insulin/IGF-1 signaling pathway in *Caenorhabditis elegans*. Front. Nutr..

[167] Lee H.-Y., Min K.-J. (2024). Betulinic Acid Increases the Lifespan of *Drosophila melanogaster* via Sir2 and FoxO Activation. Nutrients.

[168] Partridge L., Deelen J., Slagboom P.E. (2018). Facing up to the global challenges of ageing. Nature.

[169] Song S., Lam E.W.F., Tchkonia T., Kirkland J.L., Sun Y. (2020). Senescent Cells: Emerging Targets for Human Aging and Age-Related Diseases. Trends Biochem. Sci..

